# Stereoselective Ring-Opening (Co)polymerization of β-Butyrolactone and ε-Decalactone Using an Yttrium Bis(phenolate) Catalytic System

**DOI:** 10.3389/fchem.2019.00301

**Published:** 2019-05-22

**Authors:** Jiraya Kiriratnikom, Carine Robert, Vincent Guérineau, Vincenzo Venditto, Christophe M. Thomas

**Affiliations:** ^1^Department of Chemistry, Faculty of Science, Mahidol University, Bangkok, Thailand; ^2^Department of Materials Science and Engineering, School of Molecular Science and Engineering, Vidyasirimedhi Institute of Science and Technology, Rayong, Thailand; ^3^Chimie ParisTech, PSL University, CNRS, Institut de Recherche de Chimie Paris, Paris, France; ^4^CNRS UPR2301, Institut de Chimie des Substances Naturelles, Université Paris-Sud, Université Paris-Saclay, Gif-sur-Yvette Cedex, France; ^5^INSTM Research Unit, Department of Chemistry and Biology A. Zambelli, University of Salerno, Fisciano, Italy

**Keywords:** yttrium complex, ring-opening copolymerization, ε-decalactone, β-butyrolactone, tacticity

## Abstract

An effective route for ring-opening copolymerization of β-butyrolactone (BBL) with ε-decalactone (ε-DL) is reported. Microstructures of the block copolymers characterized by ^13^C NMR spectroscopy revealed syndiotactic-enriched poly(3-hydroxybutyrate) (PHB) blocks. Several di- and triblock copolymers (PDL-*b*-PHB and PDL-*b*-PHB-*b*-PDL, respectively) were successfully synthesized by sequential addition of the monomers using (salan)Y(III) complexes as catalysts. The results from MALDI-ToF mass spectrometry confirmed the presence of the copolymers. Moreover, thermal properties of the block copolymers were also investigated and showed that the microphase separation of PDL-*b*-PHB copolymers into PHB- and PDL-rich domains has an impact on the glass transition temperatures of both blocks.

## Introduction

Polyhydroxyalkanoates (PHAs) comprise a group of naturally occurring aliphatic polyesters produced by bacteria and other living organisms (Reddy et al., 2003; Tan et al., 2017). These biodegradable and hydrophobic materials combine the film-barrier properties of polyesters with the mechanical performances of petroleum-based polyethylene and polypropylene. Thanks to their interesting properties, these polymers are already used in packaging, automotive, hygienic, agricultural, and biomedical applications (Costa et al., 2013; Khosravi-Darani, 2015; Luef et al., 2015; Ali and Jamil, 2016; Yeo et al., 2018). The most common PHA is poly(3-hydroxybutyrate) (PHB), which is a linear, isotactic, high-molecular-weight polymer. However, this polymer has poor mechanical properties due to its brittleness and a susceptibility to thermal degradation slightly above its melting temperature. Therefore, development of a synthesis method producing PHBs with controlled molecular weight, lower melting temperature, and lower brittleness would be a solution for industrial manufacturing and practical use of ideal bio-based polymers (Li et al., 2016). One of the most effective approaches for the controlled synthesis of PHB is ring-opening polymerization (ROP) of β-butyrolactone (BBL) (Kricheldorf and Eggerstedt, 1997; Hori and Hagiwara, 1999; Rieth et al., 2002; Ajellal et al., 2010; Brulé et al., 2011, 2013; Li et al., 2016). ROP of β-butyrolactone mediated by metal systems has attracted much attention in recent years and had considerable achievements (Kricheldorf and Eggerstedt, 1997; Hori and Hagiwara, 1999; Rieth et al., 2002; Ajellal et al., 2010; Brulé et al., 2011). A major point of interest for some of these catalysts is the high degree of (stereo)control they exhibit under suitable conditions.

Various modification methods have been reported to improve the performance of PHB (Thomas, 2010; Aluthge et al., 2013; Lin et al., 2013; Olsén et al., 2013; Barouti and Guillaume, 2016). In particular, the formation of PHB-based copolymers might be a promising strategy but still remains a considerable synthetic challenge (Aluthge et al., 2013; Barouti and Guillaume, 2016). In this regard, poly(ε-decalactone) (PDL), a bio-based polymer synthesized by ROP of ε-decalactone (ε-DL), might be a good candidate to copolymerize with PHB in order to improve its mechanical properties (Lin et al., 2013; Olsén et al., 2013). Indeed, this polymer has been shown to be amorphous, with a low *T*_g_ value (i.e., −53°C) (Olsén et al., 2013). Although some initiators (co)polymerize ε-decalactone with good control, (Chuang et al., 2013; Lin et al., 2013; Olsén et al., 2013; Jasinska-Walc et al., 2014, 2015; Martello et al., 2014; Schneiderman et al., 2014, 2015; Lee et al., 2015; Zhu et al., 2015, 2018; Mannion et al., 2016; Tang et al., 2017) copolymerization of ε-DL with *rac*-BBL is still unknown. In this paper, we present the first example of an effective block copolymerization of BBL and DL initiated by a group 3 metal system to produce high-molecular-weight polymers with narrow molecular weight distributions.

## Results and Discussion

**Scheme 1:**
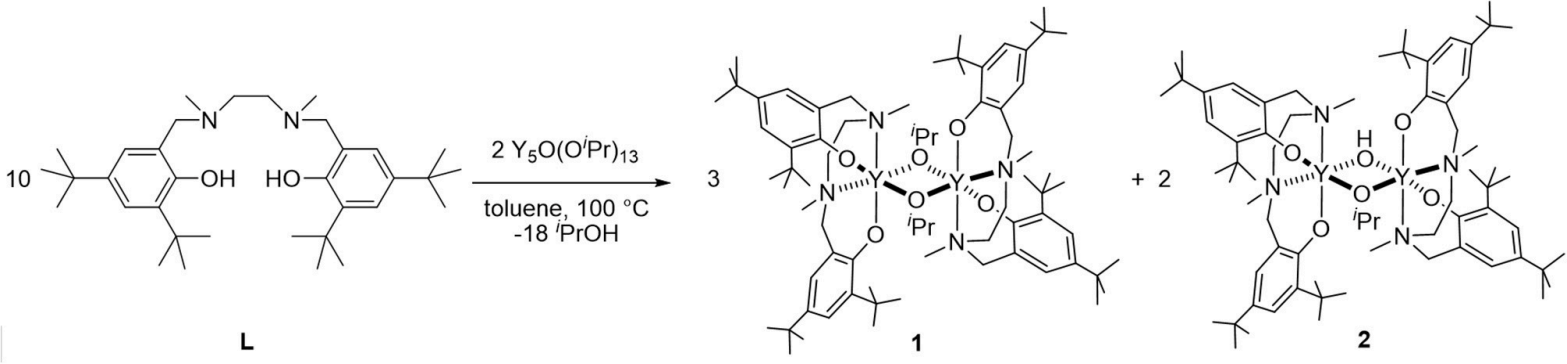
Synthesis of complexes **1** and **2**.

In our search for new copolymerization catalysts, we focused our efforts on investigating the catalytic activity of (salan)Y(III) complexes known to be active for PHB formation (Fang et al., 2013). Therefore, we decided to study the potential of such a catalytic system in a copolymerization procedure, aiming to produce PDL-PHB block copolymers with a one-pot sequential methodology. In accordance with previous observations, the reaction of commercially available Y(O^*i*^Pr)_3_ with the salan ligand **L** produces a mixture of bimetallic complexes [(**L**)Y(μ-O^*i*^Pr)]_2_ (**1**) and (**L**)_2_Y_2_(μ-O^*i*^Pr)(μ-OH) (**2**) (Scheme 1). Only volatile by-products are then removed between the yttrium alkoxide formation step and the polymerization step so that the resulting complexes are ready for polymerization without purification.

Firstly, we investigated the homopolymerization of ε-decalactone using complexes **1** and **2**. The results are summarized in Table 1. These complexes are active initiators for the controlled ROP of ε-DL under mild conditions. The corresponding polymers formed had narrow polydispersities (PDI = *M*_w_/*M*_n_), and GPC chromatograms of the isolated polymers are monomodal, suggesting that only one species in solution is active for the ROP of DL (Figure S1). As was already demonstrated for *rac*-BBL (Fang et al., 2013), we hypothesize that excess monomer cleaves the dimeric structure of both yttrium alkoxide complexes. When these species are exposed to excess DL, the monomer is coordinated and allows the formation of a mononuclear (salan)Y(O^*i*^Pr)(DL) complex and a (salan)Y(OH)(DL) species, presumably inactive for polymerization.

**Table 1:** Polymerization of ε-decalactone using (salan)Y(III) complexes^a^.

**Entry**	**[DL]/[Y]**	**[DL] (mol/L)**	**Solvent**	**Time^b^ (h)**	**Conv.^c^ (%)**	$M_{\text{n},\text{calc}}$^d^	$M_{\text{n},\text{exp}}$^d^	***M*_**w**_/$M_{\text{n}}$^d^**
1	31.3	2.5	C_6_D_6_	4	83	4,400	7,000	1.12
2	62.5	2.5	C_6_D_6_	10	92	9,800	10,500	1.26
3	62.5	1	Toluene	14	80	8,500	10,500	1.11
4	62.5	5	Toluene	14	100	10,600	11,400	1.13
5	125	–	–	20	31	6,600	7,100	1.12
6	125	2.5	C_6_D_6_	20	98	20,800	15,600	1.10
7	312.5	2.5	C_6_D_6_	50	77	41,000	14,500	1.10
8	625	2.5	C_6_D_6_	150	61	64,900	15,600	1.08

a *All reactions performed at 50°C*.

b *Time was not necessarily optimized*.

c *As determined by the integration of ^1^H NMR methine resonances of DL and PDL*.

d *M_n_ and M_w_/M_n_ of polymer determined by SEC-RI using polystyrene standards. M_n, calc_ = [DL]/[Y] × Conv. × M_DL_*.

The resulting catalytic system proved to be active at 50°C in either C_6_D_6_ or toluene solutions. For [DL]/[Y] = 62.5, polymerization in C_6_D_6_ reached 92% conversion in 10 h (Table 1, Entry 2), while polymerization in toluene achieved 80% conversion within 14 h (Table 1, Entry 3). Reducing or increasing the monomer concentration resulted in no significant change of the polymerization control (Table 1, Entries 3 and 4). Although our yttrium-based system was also active at 50°C in neat DL, low catalytic activities were observed, probably due to viscosity issues (Table 1, Entry 5) (Moritz, 1989). All other polymerization reactions were therefore preferably conducted in C_6_D_6_ at [ε-DL] = 2.5 M. Interestingly, this system proved to be active in the presence of 125–625 equiv. of lactone (Table 1, Entries 6–8), resulting in a TOF of 6 h^−1^.

For [DL]/[Y] < 100, the polymers produced have narrow molecular weight distributions and experimental number-average molecular masses (*M*_n_) close to the theoretical ones. However, for higher ratios, experimental *M*_n_ values do not correspond well with calculated *M*_n_ values. This mismatch possibly arises from suppression of hydrodynamic volume of PDL chains in THF or poor correlation between the polystyrene calibration of the GPC and the actual molecular weights of the PDL chains, as already reported by Olsén et al. (2013).

**Figure 1:**
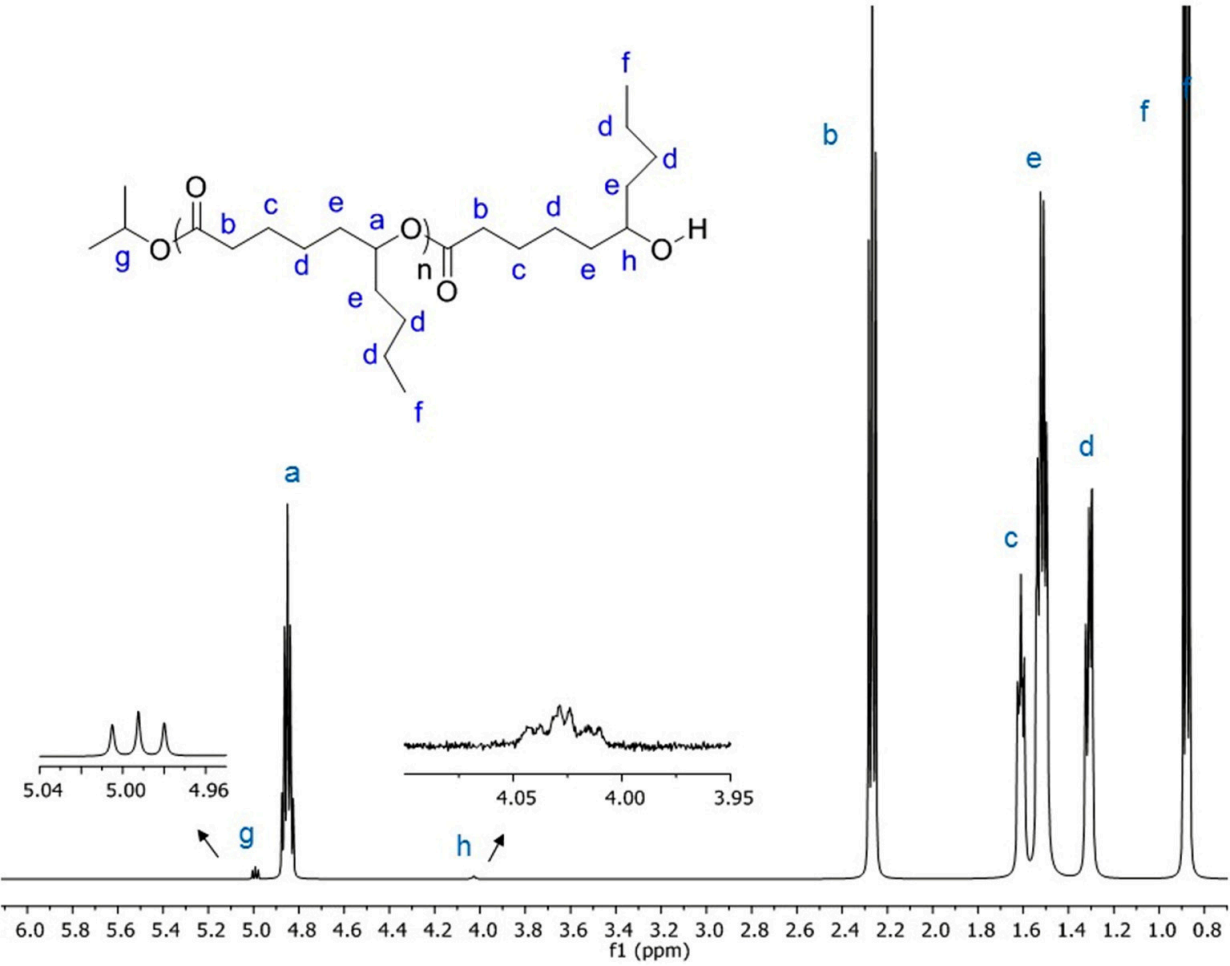
^1^H NMR (500 MHz, CDCl_3_) of PDL prepared by ROP of ε-DL with (salan)Y(III) complexes.

To confirm the mechanism, PDL was characterized by NMR spectroscopy. The results showed the polymer chain having isopropoxide and hydroxyl as end groups (Figure 1 and Figure S2), suggesting that the polymerization occurs through a coordination–insertion mechanism. The mechanism is similar to the mechanism reported in ROP of lactones or lactide catalyzed by metal alkoxide complexes (Thomas, 2010; Chuang et al., 2013; Fang et al., 2013). Matrix Assisted Laser Desorption Ionisation - Time of Flight (MALDI-ToF) mass spectrometry analysis was also performed in order to determine structure of the resulting polymer. The sample from Table 1, Entry 1, was chosen to be characterized by MALDI-ToF-MS. The spectrum reveals a repeating mass series of linear PDL having isopropoxide as an end group (O^*i*^Pr[ε-DL]_n_H + Cs^+^) (Figure S3). The result is consistent with the end-group analysis by ^1^H NMR spectroscopy. All of the PDL samples are transparent viscous liquids under ambient temperature, indicating the amorphous nature of PDL (Lin et al., 2013; Olsén et al., 2013). Having an amorphous nature, it could be a great choice to copolymerize PDL with brittle polymers to improve the performance of the resulting copolymer. Then, we investigated the catalytic activity of our yttrium system toward diblock copolymerization of ε-DL and *rac*-BBL. The copolymerization was first attempted through addition of monomer mixture to a C_6_D_6_ solution of the precursor at 60°C. However, this method failed to give block copolymers, as GPC analysis showed multimodal distributions. The copolymerization was therefore carried out by one-pot sequential copolymerization of first ε-DL and then *rac*-BBL at room temperature. The results are summarized in Table 2. For [monomers]/[Y] < 100, the copolymerization nearly reached completion for both monomers in < 5 h in C_6_D_6_ (Table 2, Entries 1 and 2). By doubling the monomer-to-metal ratio, the resultant polymer revealed a double experimental *M*_*n*_ value, indicating a controlled polymerization reaction (Table 2, Entries 1 and 2). The copolymerization was then conducted in toluene with high amounts of *rac*-BBL (Table 2, Entries 3–7). The resulting copolymers having 5–59 mol% of ε-DL were synthesized with TOF up to 44 h^−1^. Interestingly, the catalytic system also proved to be active for synthesizing PDL-*b*-PHB-*b*-PDL triblock copolymers by one-pot sequential copolymerization. The copolymerization achieved ~60% conversion of ε-DL and 99% conversion of *rac*-BBL (Table 2, Entries 8 and 9). GPC analysis of all the copolymers showed a monomodal peak with narrow molecular weight distributions *M*_w_/*M*_n_ (Figure S1). As observed for the homopolymerization of ε-DL, lower experimental *M*_n_ values were obtained, probably due to poor correlation between the polystyrene calibration of the GPC and the actual molecular weights. In order to determine the diffusion coefficients of two copolymers of different sizes (Table 2, Entries 1 and 7), DOSY NMR experiments were performed. For the low-molecular-weight copolymer sample (Table 2, Entry 1), we measured a diffusion coefficient of 2.32 × 10^−10^ m^2^.s^−1^, while the higher-molecular-weight copolymer corresponded to a diffusion coefficient of 1.88 × 10^−10^ m^2^.s^−1^ (Table 2, Entry 7). As the rate of diffusion is inversely related to the molecular weight/size, these results are consistent with GPC analyses (Figures S12, S13).

**Table 2:** Copolymerization of ε-DL and BBL using (salan)Y(III) complexes^a^.

**Entry**	**[DL+BBL+DL]/[Y]**	**ε-DL^c^ (mol%)**	**Solvent**	**Time (min)^d^**		**Conv. (%)^e^**		**$M_{\text{n},\text{exp}}$^f^**	**$M_{\text{n},\text{calc}}$^f^**	***M*_**w**_/$M_{\text{n}}$^f^**
				**ε-DL**	**BBL**	**ε-DL**	**BBL**			
1	12.5+12.5+0	46	C_6_D_6_	210	15	86	99	4,100	2,900	1.25
2	25+25+0	47	C_6_D_6_	280	10	72	82	7,600	4,800	1.25
3	10+400+0	5	Toluene	180	90	92	47	11,100	17,800	1.22
4^b^	20+380+0	8	Toluene	240	540	96	58	8,400	22,200	1.35
5^b^	40+360+0	14	Toluene	540	510	98	66	12,000	27,200	1.26
6^b^	80+320+0	25	Toluene	1,080	480	92	69	15,000	31,600	1.37
7^b^	200+200+0	59	Toluene	2,640	1,020	86	60	14,700	39,600	1.23
8	12.5+12.5+12.5	55	C_6_D_6_	330	15	61	99	4,500	3,700	1.17
9	25+25+25	51	C_6_D_6_	280	10	52	99	8,200	6,600	1.15

a *Polymerization of ε-DL and BBL were respectively performed at 50°C and room temperature with [DL] = 2.5 mol/L, unless otherwise stated*.

b *[DL+BBL] = 2.5 mol/L*.

c *DL content in copolymer*.

d *Time was not necessarily optimized*.

e *As determined by the integration of ^1^H NMR methine resonances of DL and PDL*.

f *M_n_ and M_w_/M_n_ of polymer determined by SEC-RI using polystyrene standards. M_n_ values were not corrected. M_n, calc_ = [DL]/[Y] × Conv. × M_DL_ + [BBL]/[Y] × Conv. × M_BBL_*.

**Figure 2:**
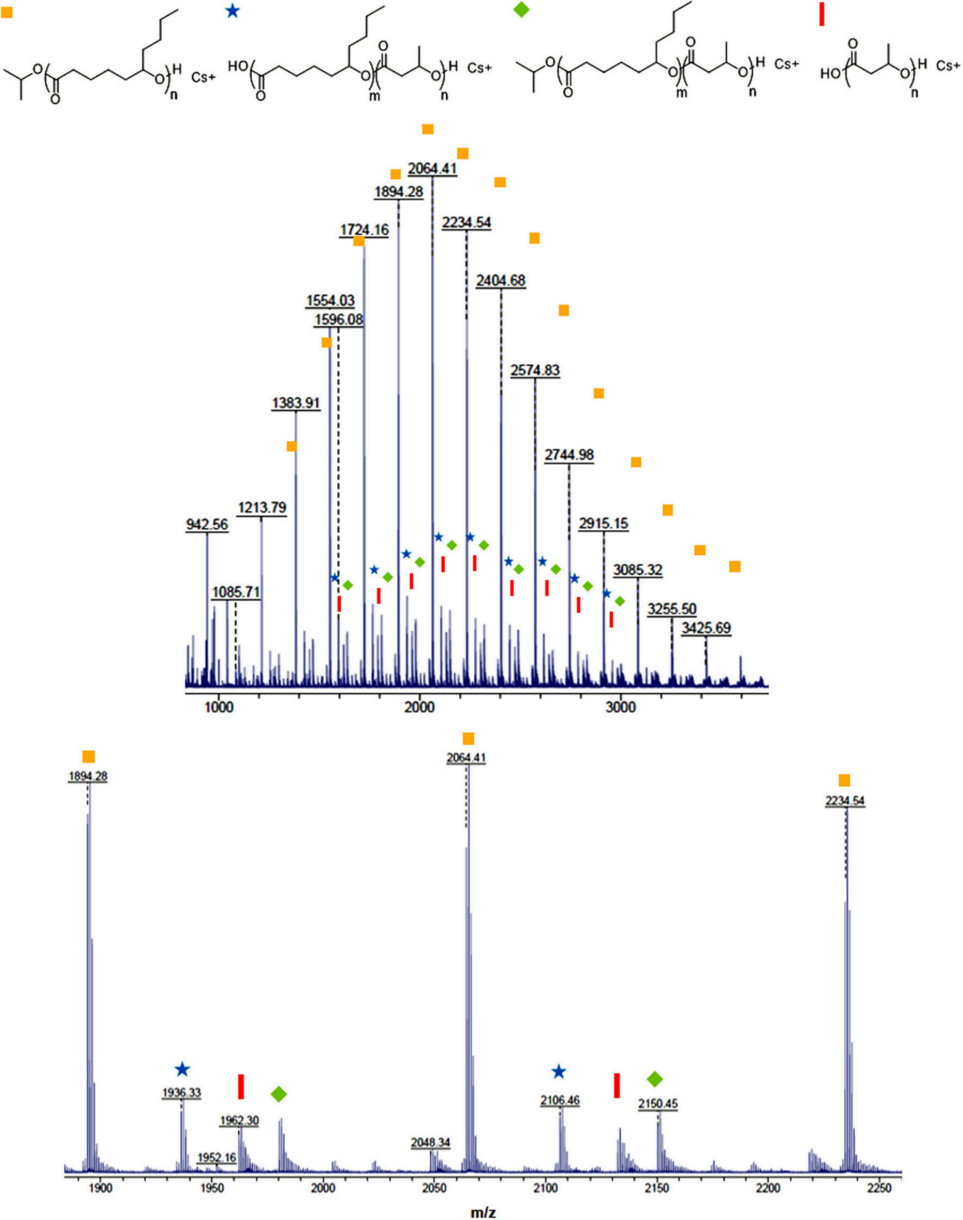
MALDI-ToF-MS spectrum of PDL-*b*-PHB copolymer synthesized by ring-opening copolymerization of ε-DL and BBL with cesium trifluoroacetate as a cationizing agent.

In order to determine topology and end groups of the block copolymers, a diblock copolymer (PDL-*b*-PHB) (Table 2, Entry 1) and a triblock copolymer (PDL-*b*-PHB-*b*-PDL) (Table 2, Entry 8) were characterized by MALDI-ToF-MS (Figure 2 and Figure S4). The highest-intensity isotope distribution corresponds to linear PDL with an isopropoxide end group. Also, analysis of the minor isotope distributions confirmed the presence of the block copolymers. For instance, in Figure 2, the peak at m/z 1,980.31 corresponds to (O^*i*^Pr[ε-DL]_10_[BBL]_1_H + Cs^+^). Similar MALDI-ToF-MS spectra showing minor series of isotope distribution of block copolymers were previously reported for poly(ε-decalactone)-*b*-poly(ω-pentadecalactone) copolymers (Jasinska-Walc et al., 2015). For PDL-*b*-PHB-*b*-PDL, similar isotope patterns are observed with mass higher than PDL-*b*-PHB (Figure S4).

### Microstructural and Statistical Analysis of the Copolymers

**Figure 3:**
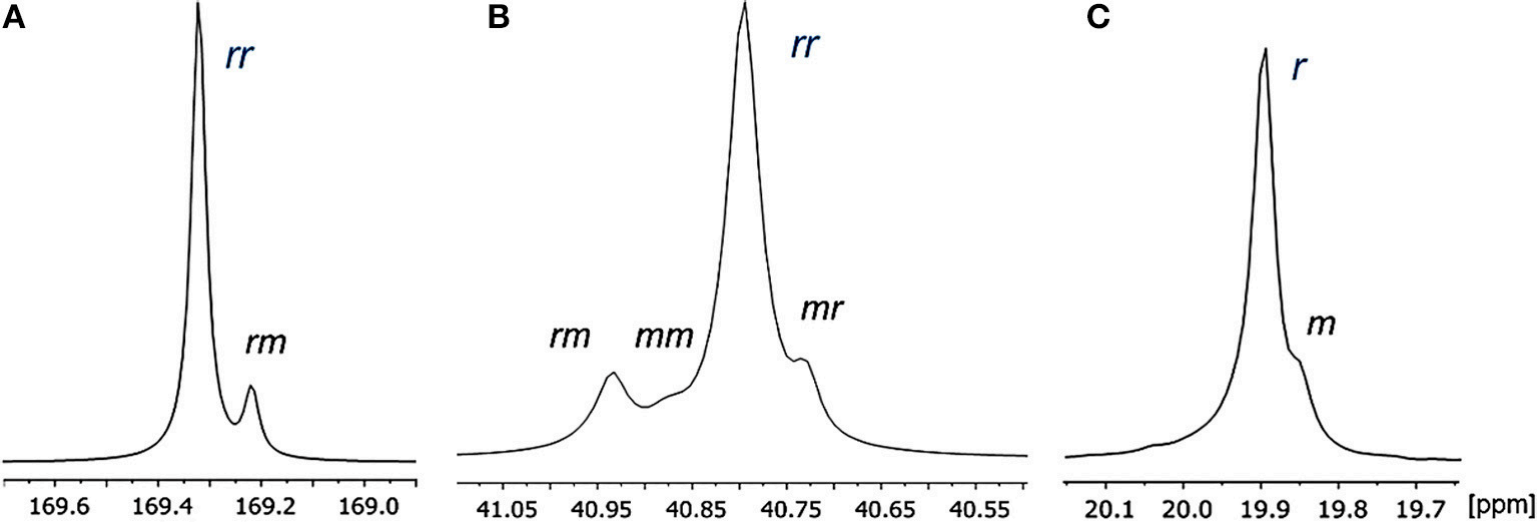
^13^C NMR spectrum (125 MHz, CDCl_3_) of **(A)** carbonyl region and **(B)** methylene region, **(C)** methyl region of PHB in PDL-*b*-PHB copolymers (Table 2, entry 6).

Microstructural analysis of PDL-*b*-PHB was studied by ^13^C NMR spectroscopy. The resonances were assigned at the diad and triad levels. Ring-opening copolymerization of ε-DL and *rac*-BBL promoted by the yttrium complexes allowed the formation of syndiotactic PHB in PDL-*b*-PHB copolymers. By comparing ^13^C NMR of the block copolymers with a prior ^13^C NMR assignment for syndiotactic PHBs (Ajellal et al., 2009a; Fang et al., 2013), expansion of both carbonyl and methylene regions of PHB in PDL-*b*-PHB copolymer showed resonances that corresponded to triad sensitivities. The most intense resonances of the carbonyl and methylene regions at δ 169.32 and δ 40.79 ppm, respectively, were correlated to *rr*-centered triads. The lower-intensity resonances at δ 169.22 (carbonyl region) and δ 40.93 ppm (methylene region) were assigned to *rm* triads, and the others at δ 40.88 and δ 40.73 (methylene region) were *mm* and *mr* triads, respectively (Figure 3).

Notably, the resonance attributed to *mm* triad is almost negligible, as expected for syndiotactic-enriched polymers (Amgoune et al., 2006; Ajellal et al., 2009a,b). The methyl region shows two resonances at the diad level. The resonance at δ 19.89 ppm was correlated to *r* diad, and the one at δ 19.84 ppm was assigned to *m* diad. Therefore, probability of racemic linkages between monomer units (*P*_r_) can be calculated (Amgoune et al., 2006; Ajellal et al., 2009a,b). As expected for syndiotactic PHBs, *P*_r_ values of PHB in PDL-*b*-PHB are high (up to 0.90 syndiotacticity), for all diblock copolymers with 5–25 mol% ε-DL (Table 3, Entries 2–5), although we observed slight differences in the PHB microstructures depending on the reaction conditions (e.g., the concentration of monomer) (Amgoune et al., 2006; Ajellal et al., 2009b; Kramer et al., 2009). Finally, the microstructure of PDL blocks was not determined, due to insufficient resolution of ε-DL resonances at 125.0 MHz (Figure S5).

**Table 3:** Thermal data of selected syndiotactic-enriched PDL-*b*-PHB copolymers obtained by using (salan)Y(III) complexes. Data of a PHB homopolymer are also reported for comparison.

**Entry**	**ε-DL^a^ (mol%)**	**PHB^b^ (wt%)**	***M*_**n, calc**_ (kDa)**	***M*_**n, GPC**_ (kDa)**	***M*_**w**_/*M*_**n**_**	**1st heating**		**Cooling**		**2nd heating**				**$P_{\text{r}}$^d^**
						***T*_**m**_ (^**°**^C)**	**Δ$H_{\text{m}}$^c^ (J/g)**	***T*_**c**_ (^**°**^C)**	**Δ$H_{\text{c}}$^c^ (J/g)**	***T*_**g**_**	***T*_**c**_ Δ*H_***c***_***	***T*_**m**_ (^**°**^C)**	**Δ$H_{\text{m}}$^c^ (J/g)**	
1	–	100	25.5	nd^e^	nd^e^	156	56	115	59	−8	–	147	61	nd^e^
2	5	91	17.8	11.1	1.22	147	59	111	52	−48, −5	–	144	59	0.84
3	8	85	22.2	8.4	1.35	152	55	110	46	−51, −2	–	148	53	0.86
4	14	75	27.2	12	1.26	152	55	110	42	−52, −1	–	145	47	0.90
5	25	60	31.6	15	1.37	155	82^c^	116	67^f^	−52, 4	–	152	73^f^	0.87

a *DL content in copolymer*.

b *Weight % of PHB block was calculated from ε**-**DL mol%*.

c *Melting and crystallization enthalpy values were calculated from the experimental data on the basis of wt% of PHB blocks (ΔH = ΔH_obs_/wt%_PHB_)*.

d *P_r_ is the probability of racemic linkages between monomer units and is determined by ^13^C{^1^H} NMR spectroscopy*.

e *nd, not determined*.

f *Value is overestimated due to possible overestimation **ε-**DL mol%*.

### Thermal and X-Ray Analyses

Differential scanning calorimetry (DSC) and wide-angle x-ray diffraction (WAXD) analyses were performed to evaluate the influence of ε-DL content on the thermal and structural properties of the copolymers. The main thermal properties [i.e., glass transition temperatures (*T*_g_), melting temperatures (*T*_m_), and melting enthalpies (Δ*H*_m_)] of the selected PDL-*b*-PHB copolymers (corresponding to polymers of Table 2, Entries 3–6) are reported in Table 3. Thermal data of a PHB sample are also shown in Table 3 for comparison. All analyzed samples crystallize from melt during the DSC cooling run, and only small differences are observed for both *T*_m_ and Δ*H*_m_ between first and second DSC heating runs. Second DSC heating thermograms of Entries 1–5 in Table 3 are reported in Figures S6–S11.

**Figure 4:**
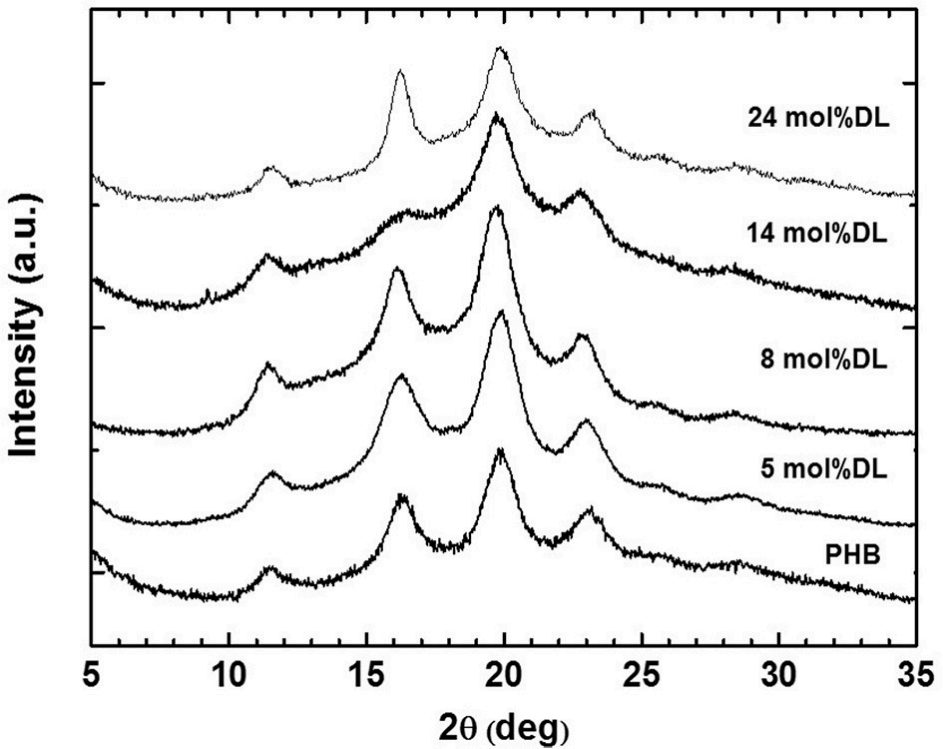
WAXD patterns of PHB homopolymer and PDL-*b*-PHB samples (entries 1–5 in Table 3) produced *via* ROP of *rac*-BBL and copolymerization of *rac*-BBL and DL with (salan)Y(III) complexes.

The detected high values of *T*_m_ (ranging from 144 to 156°C) and Δ*H*_m_ (ranging from 42 to 59 J/g) of copolymers are in good agreement with those observed for other syndiotactic-enriched PHB homopolymers (0.8 < *P*_r_ < 0.9) (Ajellal et al., 2009a; Ebrahimi et al., 2016). This hypothesis was confirmed by the WAXD patterns of all copolymers (Figure 4). Positions and intensity ratios of Bragg reflections detected in the spectra correspond to those observed for the syndiotactic PHB crystalline form (Kemnitzer et al., 1993, 1995). Moreover, WAXD spectra confirm that PDL blocks are amorphous, since no other Bragg reflection, except those of the syndiotactic PHB crystal form, was detected. Both thermal (Table 3) and structural (Figure 4) data suggest that ε-DL content ranging from 5 to 25 mol% has less effect on crystallinity of PHB blocks of PDL-*b*-PHB copolymers. It is worth noting that even by increasing ε-DL content to 59 mol% (Table 2, Entry 7), the *T*_m_ of the PHB block remains high (i.e., 143°C). Based on these data, it appears that the PDL block, probably due to its high flexibility, has no influence on the ability of the PHB block to crystallize.

**Figure 5:**
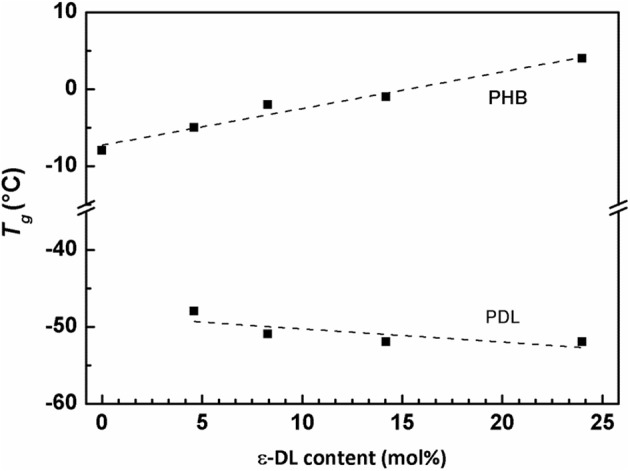
Plot of *T*_*g*_(second run) vs. ε-DL mol% content of polymers from Table 3 (entries 1–5).

The observed small differences in *T*_m_ of copolymers reported in Table 3 are possibly due to small differences in stereoregularity of the BBL unit sequences rather than to the PDL block lengths (Figure S14). These differences could explain why, in the second DSC heating run, the copolymer with 25 mol% of DL units (Table 3, Entry 5) has a *T*_*m*_ higher than the one of the PHB homopolymer having similar *M*_n_ (Table 3, Entry 1). In addition, PDL-*b*-PHB copolymers exhibited two *T*_g_ values (*T*_g_ values were evaluated by second DSC heating runs and reported in Table 3), one in the range −5, +4°C, similar to the *T*_g_ of a PHB homopolymer (Ajellal et al., 2009a), and the second in the range −52, −48°C, comparable to the *T*_g_ of a PDL homopolymer (Olsén et al., 2013). This probably results in the immiscibility of PHB and PDL blocks (Olsén et al., 2013). In Figure 5, *T*_g_ of both PDL and PHB blocks of the polymers reported in Table 3 was plotted as a function of ε-DL mol% content. A roughly linear behavior for both glass transition temperatures is observed. Therefore, increasing the length of the PDL blocks increases the glass transition temperatures of the PHB blocks while slightly decreasing the ones of the PDL blocks.

The behavior of both glass transition temperatures does not match the typical Flory–Fox (Fox and Flory, 1950) behavior, which, for low *M*_*n*_ values,^1^ predicts the *T*_g_ increase with increasing *M*_*n*_. For the PDL block, the *T*_g_ decreases from −48 to −52°C when *M*_n, calc_ increases from 1.6 to 12.5 kDa. For the PHB block, copolymers (Table 3, Entries 2–5) have *T*_g_ ranging from 4 to −5°C, while the homopolymer (Table 3, Entry 1) has both the lowest *T*_g_ (−8°C) and the highest *M*_n_,_calc_ (25.5 kDa). This behavior can be explained assuming that PDL-*b*-PHB copolymers are constituted by immiscible blocks arranged in separated microphases. Based on this assumption, the reduction of the volume fraction^2^ of the PHB phase (*f*_PHB_), which decreases from 0.9 to 0.55 with increasing ε-DL content from 5 to 25 mol%, corresponds to a size decrease of PHB-rich domains. Moreover, we can also assume that the PHB blocks have a decreasing mobility, as they are segregated in gradually decreasing space and surrounded by an immiscible phase. As it is generally accepted that *T*_g_ is inversely related to polymer flexibility and mobility, the observed increase of *T*_g_ of PHB can therefore be explained by the reduction of the PHB block mobility due to the microphase separation of PDL-*b*-PHB copolymers into PDL and PHB domains.

For the PDL block, with increasing volume fraction of the PDL phase (*f*_PDL_), which increases from 0.1 to 0.45 with increasing ε-DL content from 5 to 25 mol%, the size of PDL-rich domains, increases and the mobility of PDL blocks, segregated into increasing space, is assumed to increase. Consequently, with increasing *f*_PDL_, the PDL block mobility increases approaching the mobility of PDL homopolymer, and as a result, *T*_g_ of the PDL block approaches that of the homopolymer (i.e., −53°C) (Olsén et al., 2013).

## Conclusion

We have reported for the first time the ring-opening copolymerization of ε-DL with *rac*-BBL catalyzed by an yttrium-based catalytic system. Di- and triblock copolymers, PDL-*b*-PHB and PDL-*b*-PHB-*b*-PDL, were synthesized by means of one-pot sequential polymerization of ε-DL and *rac*-BBL. In agreement with NMR observations, thermal and structural analyses of PDL-*b*-PHB copolymers suggested that the observed crystallinity is due to the syndiotactic PHB block. Our results demonstrated that the PHB block crystallizes even in the presence of long, amorphous, and highly flexible PDL blocks. Moreover, it has been also observed that the microphase separation of PDL-*b*-PHB copolymers into PHB- and PDL-rich domains has an impact on the glass transition temperature of both blocks, which is not in agreement with the Flory–Fox relationship. As observed for a thermoplastic elastomer, the physical behavior of these copolymers (i.e., stiffness/flexibility) can be tuned by changing the ε-DL/BBL ratio, thanks to the simultaneous presence of a highly flexible phase (PDL block) associated with a rigid crystalline phase (PHB block). Such tunable physical behavior makes these copolymers industrially relevant.

## Author Contributions

JK, VG, and CR performed the experiments. CT, JK, and VV wrote the manuscript with support from the co-authors. All authors analyzed the data, discussed the results, and commented on the manuscript.

### Conflict of Interest Statement

The authors declare that the research was conducted in the absence of any commercial or financial relationships that could be construed as a potential conflict of interest.

## Footnotes

^1^More correctly, for *T*_*g*_ values lower than *T*_*g*_ of polymer with infinite molecular mass. *T*_*g*_ of a shortly branched polymer with *M*_n_ > 20–30 kDa is commonly considered to be independent of *M*_n_.

^2^The volume fraction (*f*), at different ε-DL mol%, was calculated by using *M*_*n*.*calc*_ of both polymer blocks and density values of 0.97 and 1.22 g/cm^3^ for PDL and PHB blocks, respectively.
