# Supplementary material for: Stereoselective Ring-Opening (Co)polymerization of β-Butyrolactone and ε-Decalactone Using an Yttrium Bis(phenolate) Catalytic System
Source: Front Chem. 2019 May 22;7:301. doi: 10.3389/fchem.2019.00301 (PMC6541034; doi:10.3389/fchem.2019.00301)
Supplement: Supplementary file 1 [file Data_Sheet_1.pdf]

Supporting Information

Stereoselective Ring-Opening (Co)polymerization of β-butyrolactone and ε-Decalactone using an Yttrium Bis(phenolate) Catalytic System

Jiraya Kiriratnikom,[a,b,c] Carine Robert,[c] Vincent Guérineau,[d] Vincenzo Venditto,[*,e] and Christophe M. Thomas[*,c]

General

General considerations. All operations were carried out under dry argon atmosphere using standard Schlenk techniques or in a glovebox Jacomex [GP]concept with O_2 and H_2O purification system. Toluene was taken from a solvent purification system (PureSolv, Innovative technology Inc.), dried and distilled over Na/benzophenone under argon and degassed thoroughly by freeze-vacuum-thaw cycles prior to use. Deuterated benzene (Eurisotop) was dried and freshly distilled over Na/benzophenone under argon and degassed prior to use. (salan)Y(III)complexes **1** and **2** were synthesized following a literature procedure.[1] Racemic β-butyrolactone (*rac*-BBL) (Aldrich) and ε-decalactone (ε-DL) (Aldrich) were purified by distillation over calcium hydride under argon and degassed prior to use. Other chemicals were purchased from commercial supplier and were used as received.

Measurements.

^{1}H and $^{13}C\{^{1}H\}$ NMR spectra were recorded on a Bruker Avance 400 MHz spectrometer or a Bruker AC 500 MHz spectrometer and referenced to residual signal of chloroform-*d* ($CDCl_3$, δ 7.26 ppm, 77.16 ppm) or benzene-d_6 (C_6D_6, δ 7.16 ppm, 128.06 ppm) as internal standards for ^{1}H and $^{13}C\{^{1}H\}$ NMR, respectively.

MALDI-ToF mass spectrometry analyses were performed using an UltrafleXtreme mass spectrometer (Bruker Daltonics, Bremen) and a Axima Confidence spectrometer (Shimadzu). Acquisitions were performed in reflecton or linear positive ion mode. The laser intensity was set just above the ion generation threshold to obtain peaks with the highest possible signal-to-noise (S/N) ratio without significant peak broadening. The mass spectrometer was externally calibrated using PEG4500. All data were processed using the program FlexAnalysis (Bruker Daltonics,

Bremen) or Axima program (Shimadzu). Polymer sample for MALDI analysis was prepared at a concentration of 60 μM in THF. The matrix solution was prepared at a concentration of 6 mM in THF. The cationizing agent, cesium trifluoroacetate or potassium trifluoroacetate was prepared at 0.7 mM in THF. The sample was prepared by mixing the polymer solution with matrix solution and cationizing agent solution at a volume ratio of 1:9:1.

Size exclusion chromatography (SEC) was performed in THF at 35 °C using an Agilent 1260 Infinity Series GPC (ResiPore 3 μm, 300 ×7.5 mm, 1.0 mL min-1, UV (250 nm) and RI (PLGPC 220) detectors. Molecular weights and molecular weight distributions of the resultant polymers were calculated with reference to a universal calibration *vs.* polystyrene standards (limits M_w = 200 to 400 000 g mol^{-1}). M_n values were not corrected.

Differential scanning calorimetry (DSC) data were obtained at Department of Chemistry and Biology of the University of Salerno (Italy) by using a Q20 TA Instruments apparatus, calibrated with indium. Measurements were performed on "as polymerized" (as it is obtained by polymerization process) samples under nitrogen flow with a heating/cooling rate of 10 °C/min in the range of −40 to +240 °C. DSC data were processed with TA Universal Analysis v2.3 software. TGA data were obtained by using a thermo balance SDT Q600 TA Instruments. The analysis was carried out under nitrogen flow (100 cm^3/min STP) in the range 20 - 800 °C at 10 °C/min heating rate. Measurements were performed both on as polymerized and dichloromethane casting film samples. Wide-Angle X-ray Diffraction patterns of as polymerized polymer samples were obtained with an automatic Bruker D8 diffractometer. Measurements were performed by using a nickel-filtered Cu Kα radiation (λ = 1.5418 Å) and a Vantec PSD detector.

Typical procedure for ε-DL Homopolymerization. In the glove box, a Schlenk tube was charged with a solution of the initiator (19.5 mg, $2.36x10^{-5}$ mol of active initiator) and ε-DL (0.25 g, 1.47 mmol, 62.5 equiv. per active initiator) in 0.5 mL benzene-d_6. The reaction solution was stirred at 50°C for the desired time. Conversion was monitored using ^{1}H NMR spectroscopy by comparing the relative magnitude of peaks corresponding to the methine hydrogen for ε-DL and polydecalactone (PDL). The reaction mixture was stopped by opening the Schlenk tube to air atmosphere and the polymer was precipitated with excess pentane. The polymer was collected and dried under vacuum to constant weight.

Typical copolymerization reaction for diblock copolymers. In the glove box, a Schlenk tube was charged with a solution of the initiator (82.0 mg, $9.90x10^{-5}$ mol of active initiator) and ε-DL (0.21 g, 1.25 mmol, 12.5 equiv. per active initiator) in 0.5 mL benzene-d_6. The reaction solution was stirred at 50°C for the desired time. Conversion was monitored using ^{1}H NMR ^{1}by comparing the relative magnitude of peaks corresponding to the methine hydrogen for ε-DL and poly(ε-decalactone) (PDL). Then, BBL (0.11 g, 1.25 mmol, 12.5 equiv. per active initiator) was added to the solution mixture. The mixture was stirred for the desired time at room temperature. Conversion was monitored using ^{1}H NMR spectroscopy by comparing the relative magnitude of peaks corresponding to the methine hydrogen for BBL and PHB. At the end of the reaction, the reaction was stopped by opening the Schlenk tube to air atmosphere and the copolymer was precipitated with excess pentane. The copolymer was collected and dried under vacuum to constant weight.

Typical copolymerization reaction for triblock copolymers. In the glove box, a Schlenk tube was charged with a solution of the initiator (41.0 mg, $4.96x10^{-5}$ mol of active initiator) and ε-DL (0.21g, 1.25 mmol, 25 equiv. per active initiator) in 0.5 mL benzene-d_6. The reaction solution was

stirred at 50ºC for the desired time. Conversion was monitored by ^{1}H NMR by comparing the relative magnitude of peaks corresponding to the methine hydrogen for ε-DL and PDL. Then, BBL (0.11 g, 1.25 mmol, 25 equiv. per active initiator) was added to the solution mixture. The mixture was stirred for the desired time at room temperature. Conversion was monitored by ^{1}H NMR by comparing the relative magnitude of peaks corresponding to the methine hydrogen for BBL and PHB. At the end of the reaction period, ε-DL (0.21 g, 1.25 mmol, 25 equiv. per active initiator) was added and the reaction mixture was then stirred at 50ºC for the additional desired time. The progress of the reaction was followed by ^{1}H NMR. The reaction was stopped by opening the Schlenk tube to air atmosphere and the copolymer was precipitated with excess pentane. The copolymer was collected and dried under vacuum to constant weight.

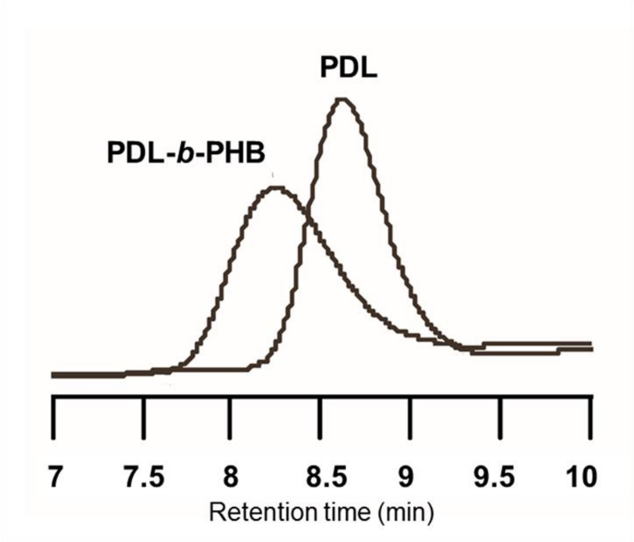

Figure S1. GPC traces (THF, 35°C, RI detection) of PDL (Table 1, Entry 1) and PDL-*b*-PHB copolymer (Table 2, Entry 5).

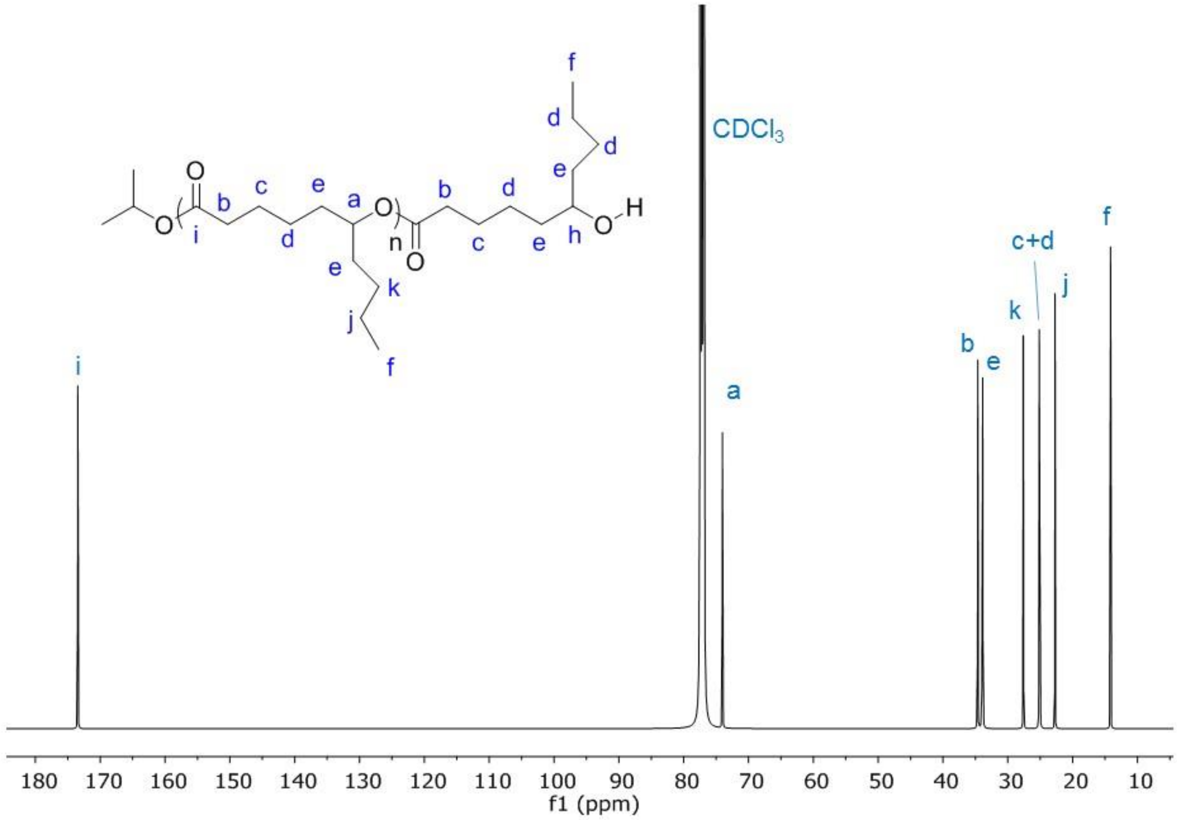

Figure S2. ^{13}C NMR (125MHz, $CDCl_3$) of PDL prepared by ROP of ε-DL with (salan)Y(III) complexes.

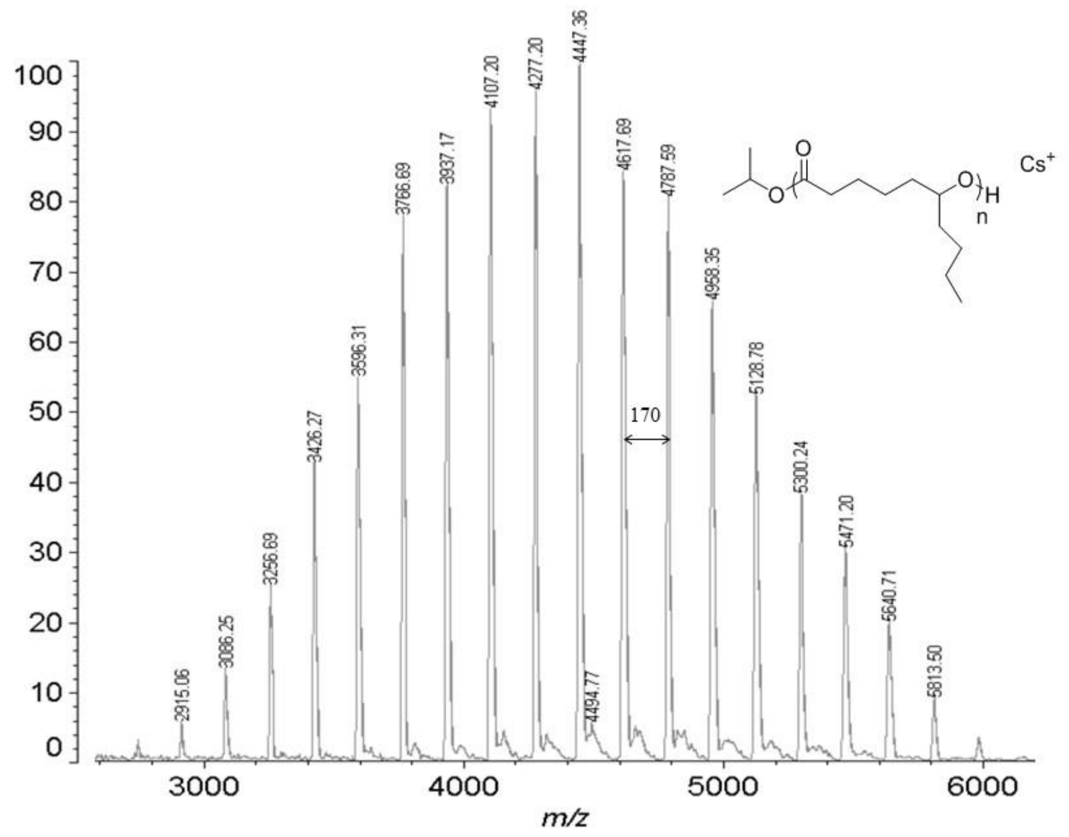

Figure S3. MALDI-ToF-MS spectrum of PDL prepared by ROP of ε-DL with (salan)Y(III) complexes; [DL]/[Y] = 31.3:1 at 50ºC (Table 1, Entry 1) showing a series of $O^{i}Pr[\varepsilon\text{-DL}]_{n}H + Cs^{+}$

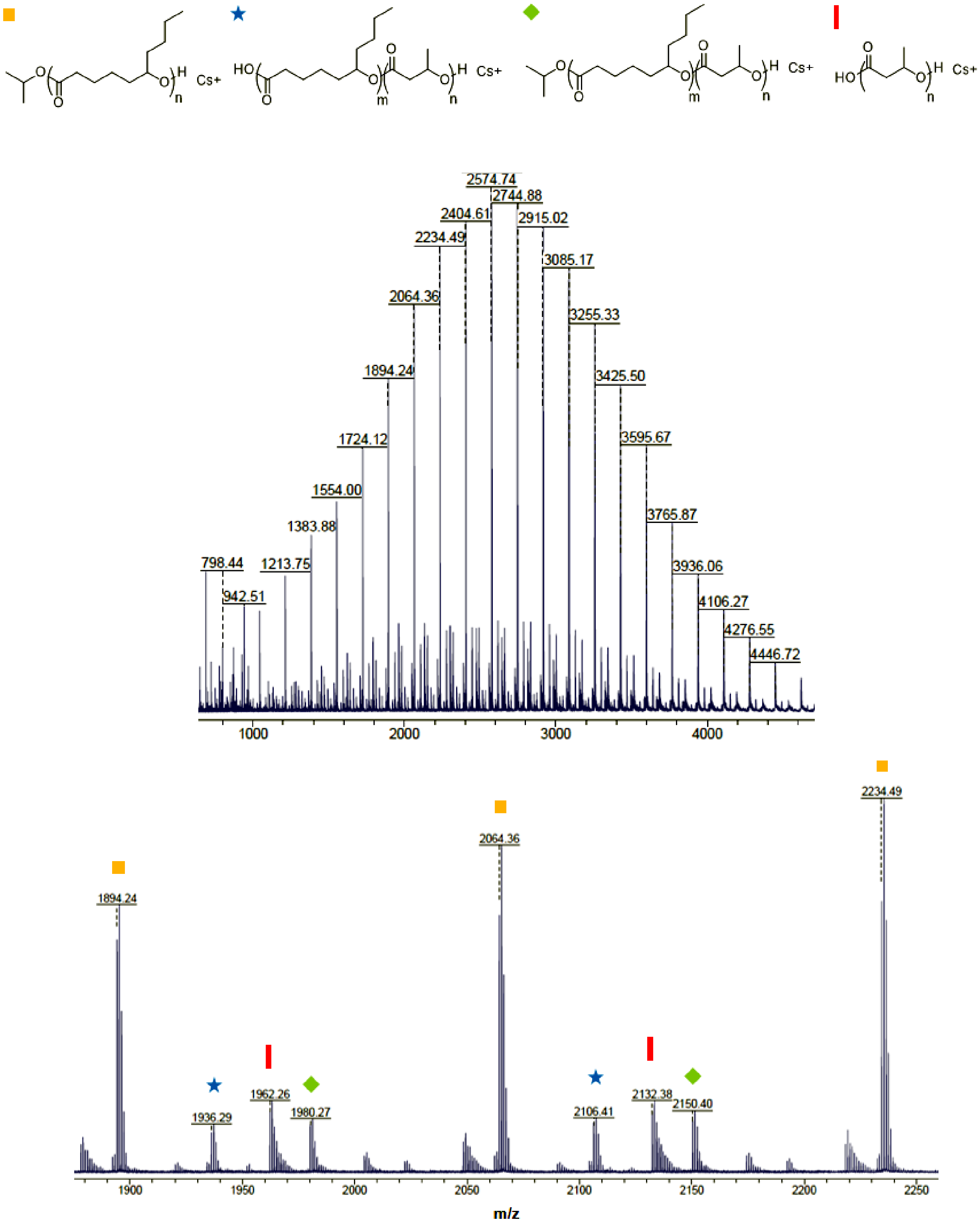

Figure S4. MALDI-ToF-MS spectrum of PDL-*b*-PHB-*b*-PDL copolymer synthesized by ring-opening copolymerization of ε-DL and BBL with cesium trifluoroacetate as a cationizing agent.

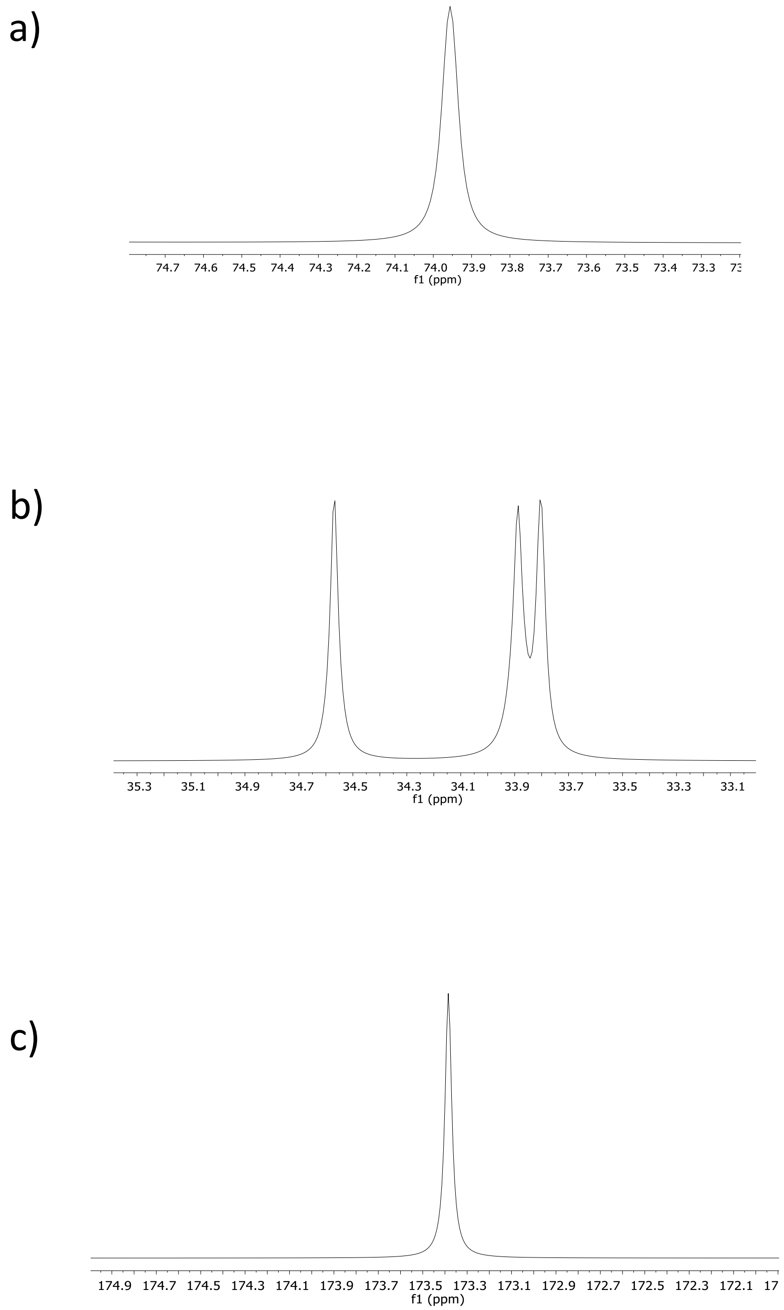

Figure S5. ^{13}C{H} NMR spectrum (125MHz, $CDCl_3$) of (a) methine, (b) methylene, (c) carbonyl regions of PDL prepared by ROP of ε-DL with (salan)Y(III) complexes.

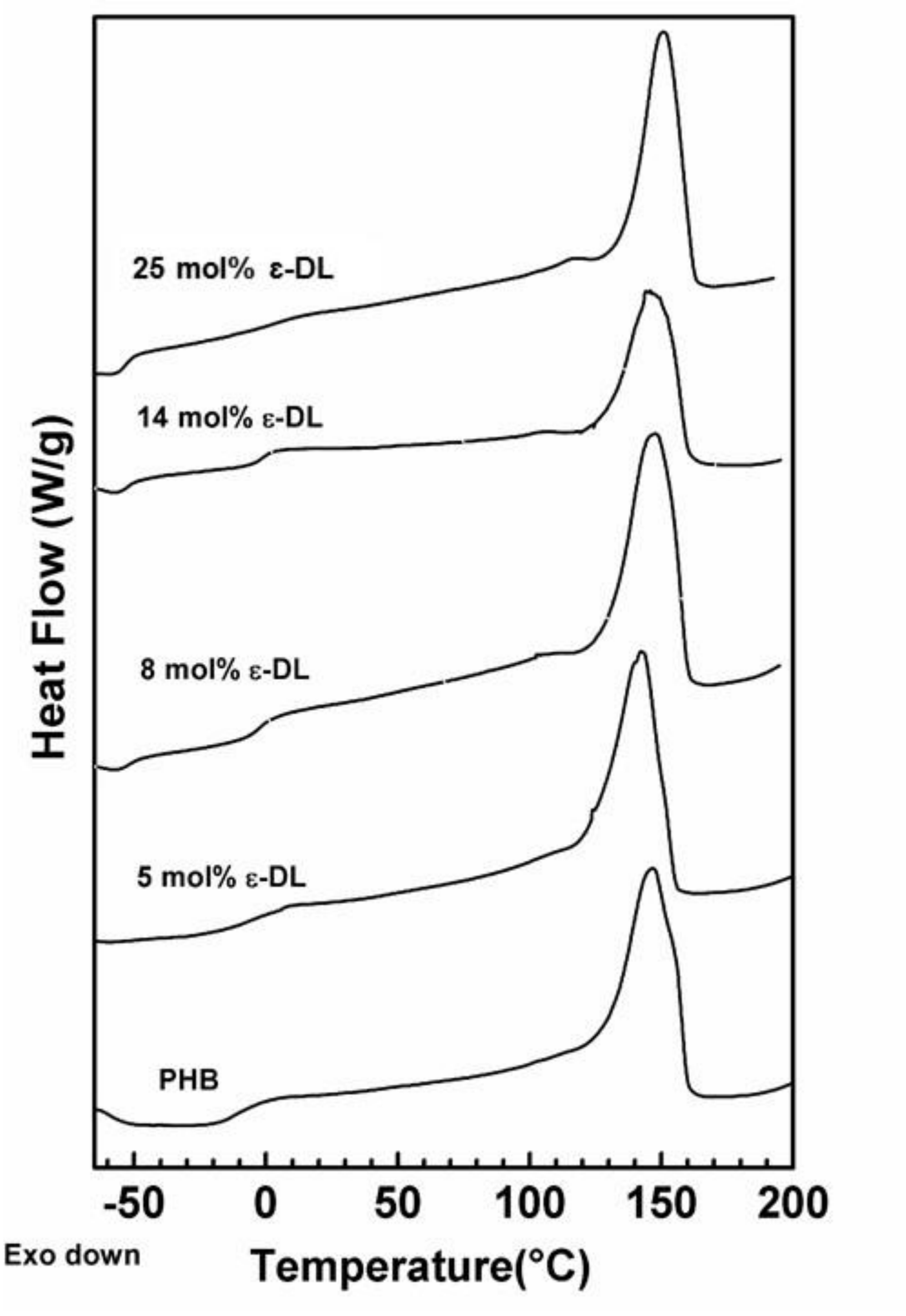

Figure S6. DSC traces (second run) of (co)polymers prepared with (salan)Y(III) complexes (Table 3, Entries 1-5).

a

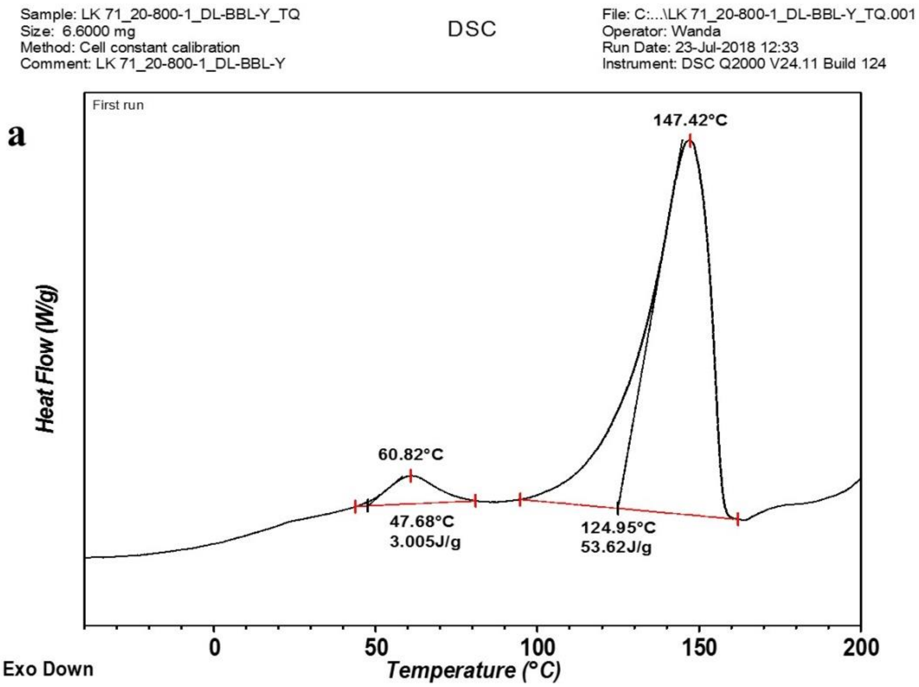
Sample: LK 71_20-800-1_DL-BBL-Y_TQ
Size: 6.6000 mg
Method: Cell constant calibration
Comment: LK 71_20-800-1_DL-BBL-Y
DSC
File: C:...\LK 71_20-800-1_DL-BBL-Y_TQ.001
Operator: Wanda
Run Date: 23-Jul-2018 12:33
Instrument: DSC Q2000 V24.11 Build 124
First run
147.42°C
60.82°C
47.68°C
3.005J/g
124.95°C
53.62J/g
Heat Flow (W/g)
0
50
100
150
200
Exo Down
Temperature (°C)

b

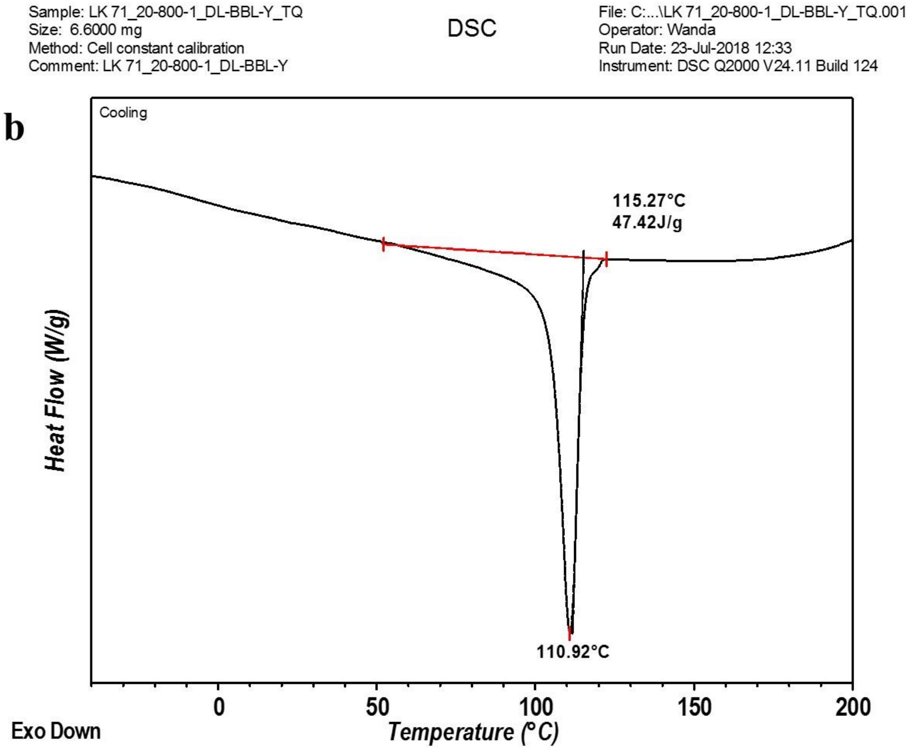
Sample: LK 71_20-800-1_DL-BBL-Y_TQ
Size: 6.6000 mg
Method: Cell constant calibration
Comment: LK 71_20-800-1_DL-BBL-Y
DSC
File: C:...\LK 71_20-800-1_DL-BBL-Y_TQ.001
Operator: Wanda
Run Date: 23-Jul-2018 12:33
Instrument: DSC Q2000 V24.11 Build 124
Cooling
115.27°C
47.42J/g
110.92°C
Heat Flow (W/g)
0
50
100
150
200
Exo Down
Temperature (°C)

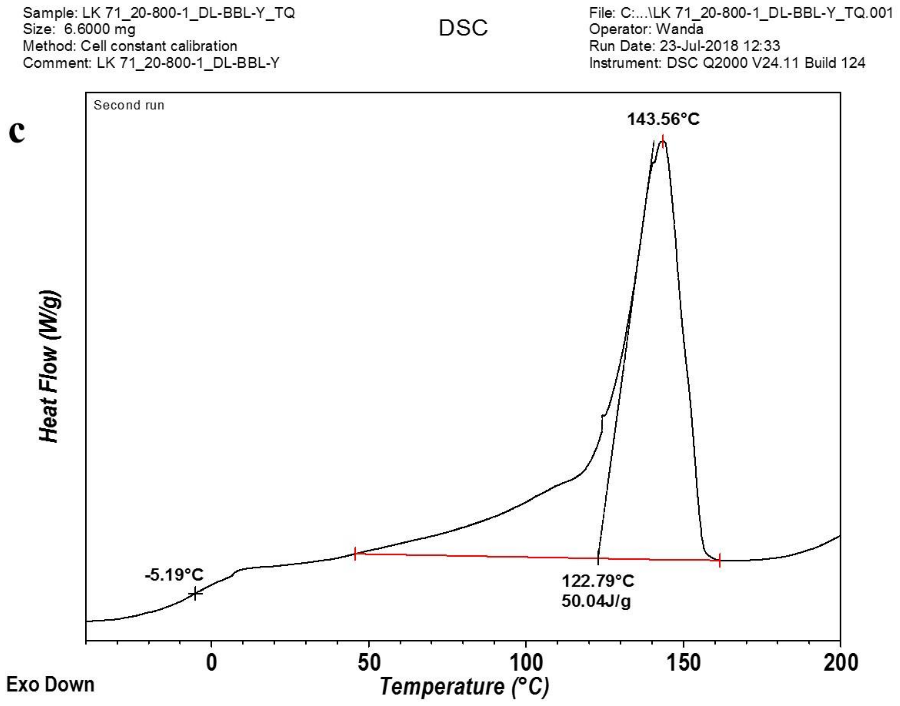

Figure S7. DSC trace a) first run b) cooling c) second run of PDL-*b*-PHB (5 mol% DL) prepared by copolymerization of ε-DL and *rac*-BBL with (salan)Y(III) complexes (Table 3, Entry 2).

a

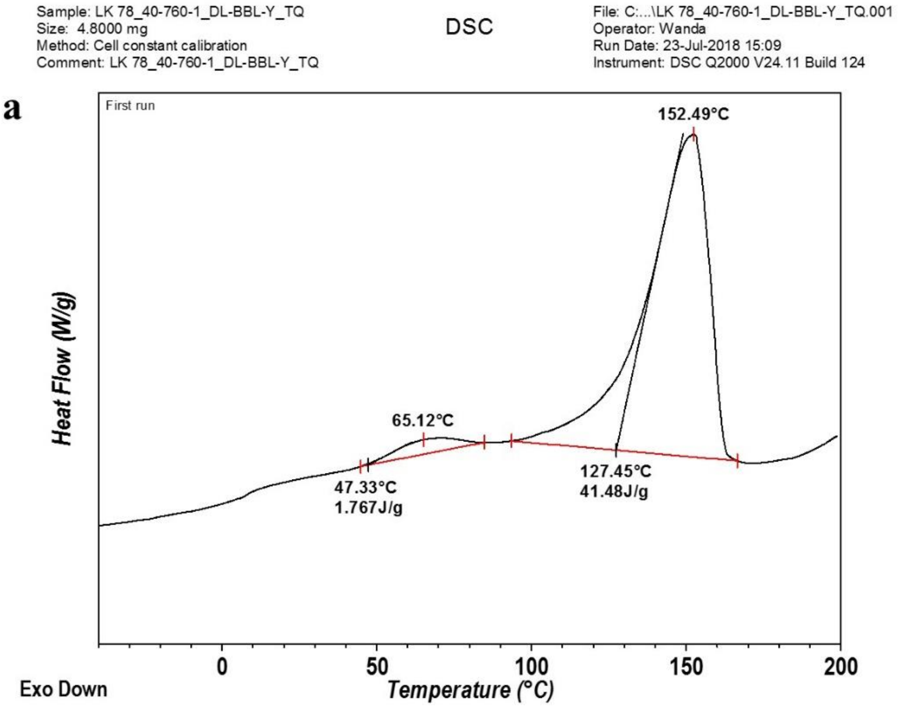

Sample: LK 78_40-760-1_DL-BBL-Y_TQ
Size: 4.8000 mg
Method: Cell constant calibration
Comment: LK 78_40-760-1_DL-BBL-Y_TQ
DSC
File: C:...\LK 78_40-760-1_DL-BBL-Y_TQ.001
Operator: Wanda
Run Date: 23-Jul-2018 15:09
Instrument: DSC Q2000 V24.11 Build 124
First run
152.49°C
65.12°C
47.33°C
1.767J/g
127.45°C
41.48J/g
Heat Flow (W/g)
0
50
100
150
200
Exo Down
Temperature (°C)

b

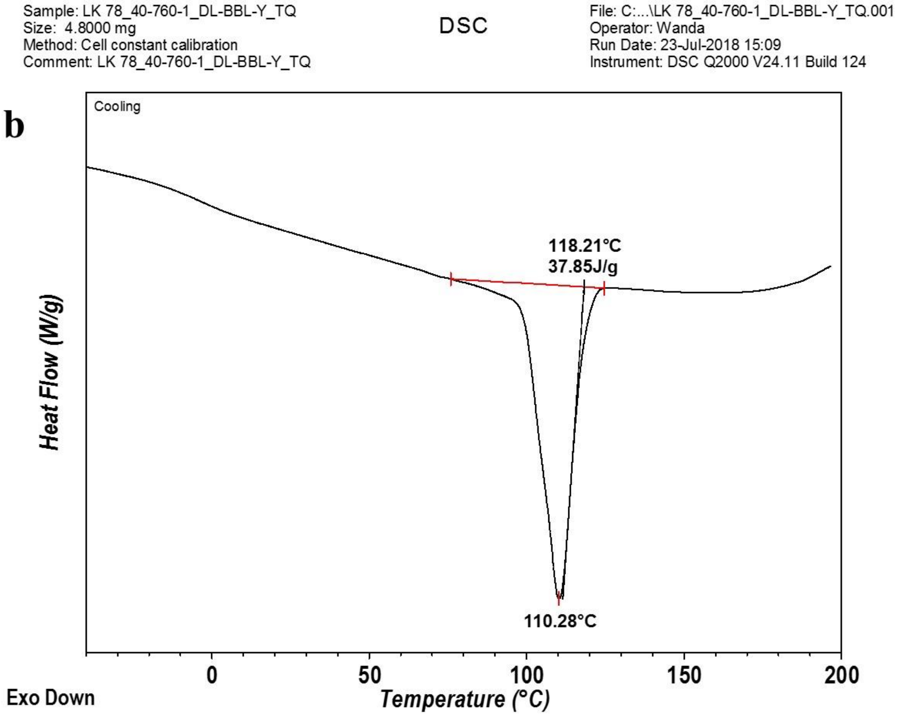

Sample: LK 78_40-760-1_DL-BBL-Y_TQ
Size: 4.8000 mg
Method: Cell constant calibration
Comment: LK 78_40-760-1_DL-BBL-Y_TQ
DSC
File: C:...\LK 78_40-760-1_DL-BBL-Y_TQ.001
Operator: Wanda
Run Date: 23-Jul-2018 15:09
Instrument: DSC Q2000 V24.11 Build 124
Cooling
118.21°C
37.85J/g
110.28°C
Heat Flow (W/g)
0
50
100
150
200
Exo Down
Temperature (°C)

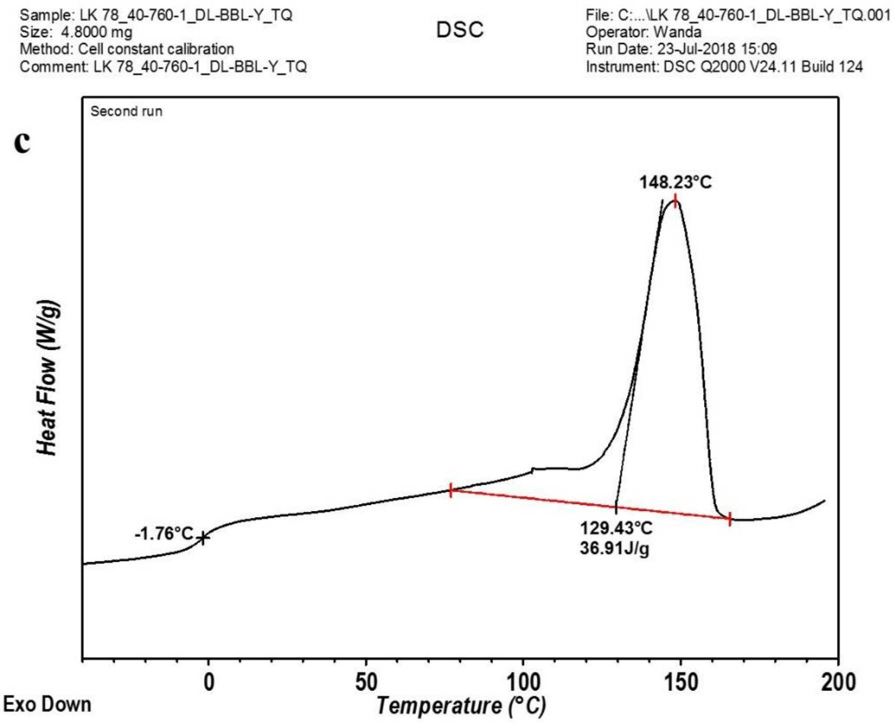

Figure S8. DSC trace a) first run b) cooling c) second run of PDL-*b*-PHB (8 mol% DL) prepared by copolymerization of ε-DL and *rac*-BBL with (salan)Y(III) complexes (Table 3, Entry 3).

a

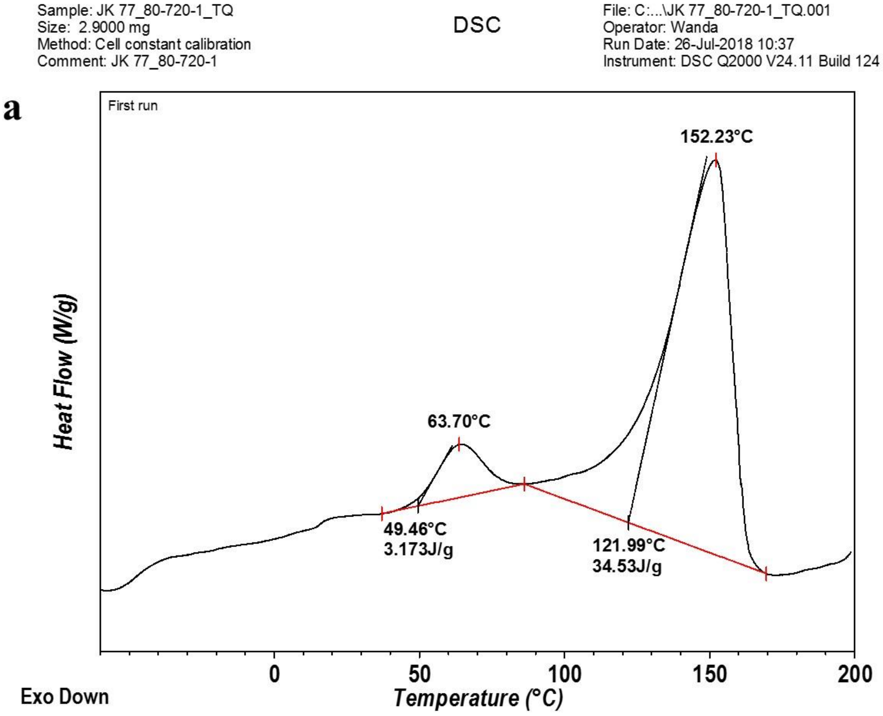
Sample: JK 77_80-720-1_TQ
Size: 2.9000 mg
Method: Cell constant calibration
Comment: JK 77_80-720-1
DSC
File: C:...\JK 77_80-720-1_TQ.001
Operator: Wanda
Run Date: 26-Jul-2018 10:37
Instrument: DSC Q2000 V24.11 Build 124
First run
152.23°C
63.70°C
49.46°C
3.173J/g
121.99°C
34.53J/g
Heat Flow (W/g)
0
50
100
150
200
Exo Down
Temperature (°C)

b

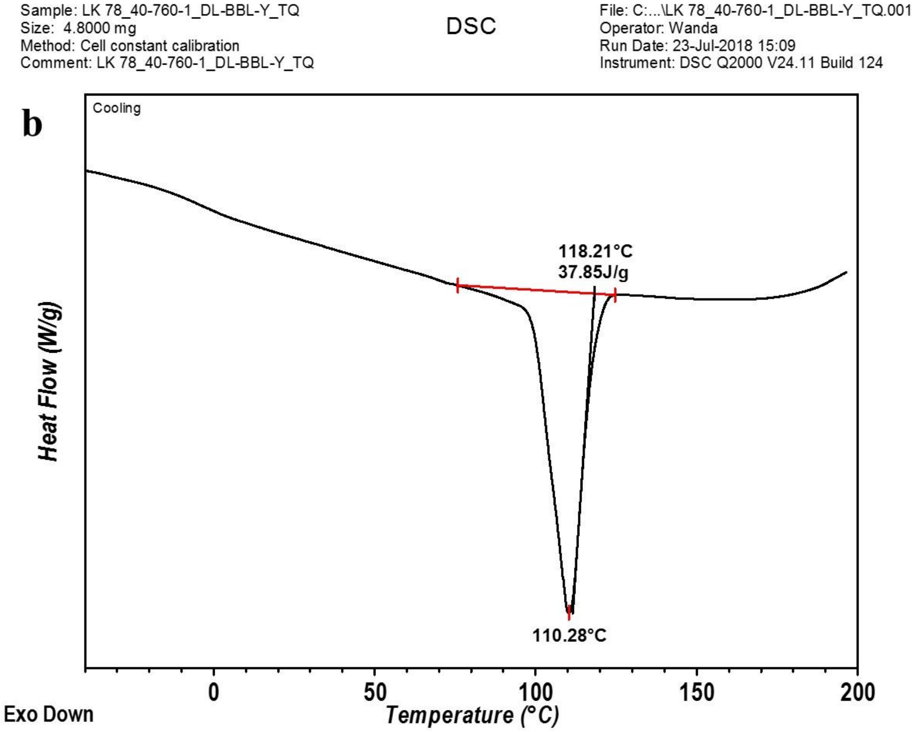
Sample: LK 78_40-760-1_DL-BBL-Y_TQ
Size: 4.8000 mg
Method: Cell constant calibration
Comment: LK 78_40-760-1_DL-BBL-Y_TQ
DSC
File: C:...\LK 78_40-760-1_DL-BBL-Y_TQ.001
Operator: Wanda
Run Date: 23-Jul-2018 15:09
Instrument: DSC Q2000 V24.11 Build 124
Cooling
118.21°C
37.85J/g
110.28°C
Heat Flow (W/g)
0
50
100
150
200
Exo Down
Temperature (°C)

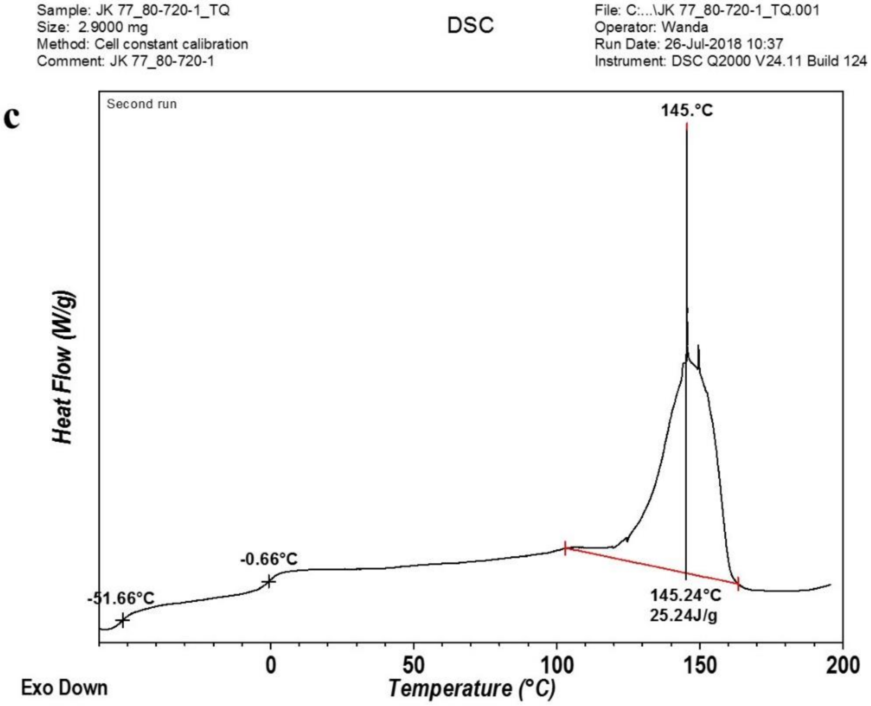

Figure S9. DSC trace a) first run b) cooling c) second run of PDL-*b*-PHB (14 mol% DL) prepared by copolymerization of ε-DL and *rac*-BBL with (salan)Y(III) complexes (Table 3, Entry 4).

a

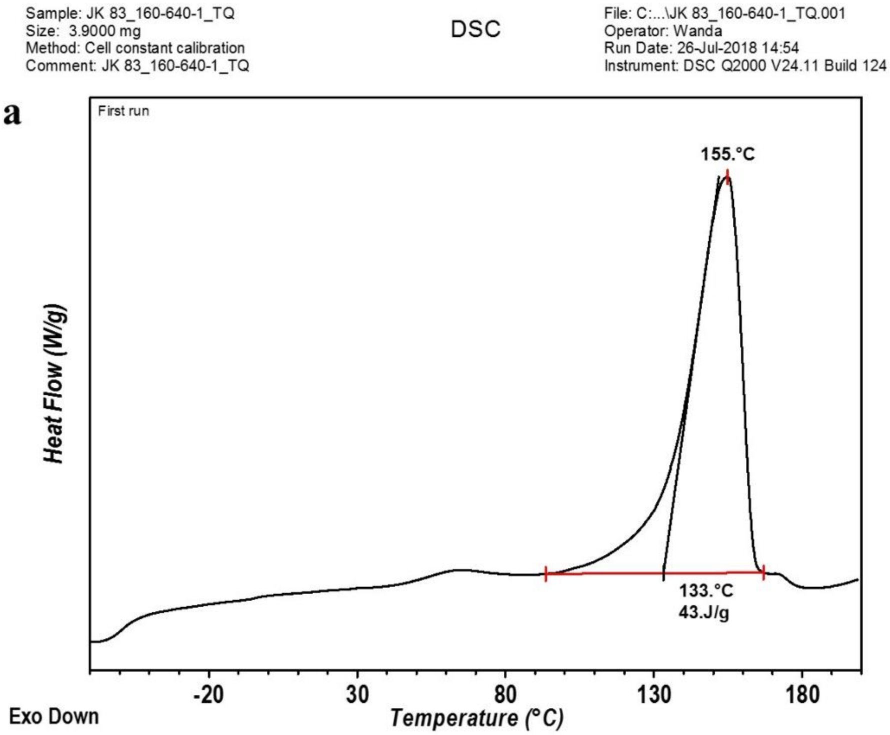
Sample: JK 83_160-640-1_TQ
Size: 3.9000 mg
Method: Cell constant calibration
Comment: JK 83_160-640-1_TQ
DSC
File: C:...\JK 83_160-640-1_TQ.001
Operator: Wanda
Run Date: 26-Jul-2018 14:54
Instrument: DSC Q2000 V24.11 Build 124
First run
155.°C
133.°C
43.J/g
Heat Flow (W/g)
-20
30
80
130
180
Exo Down
Temperature (°C)

b

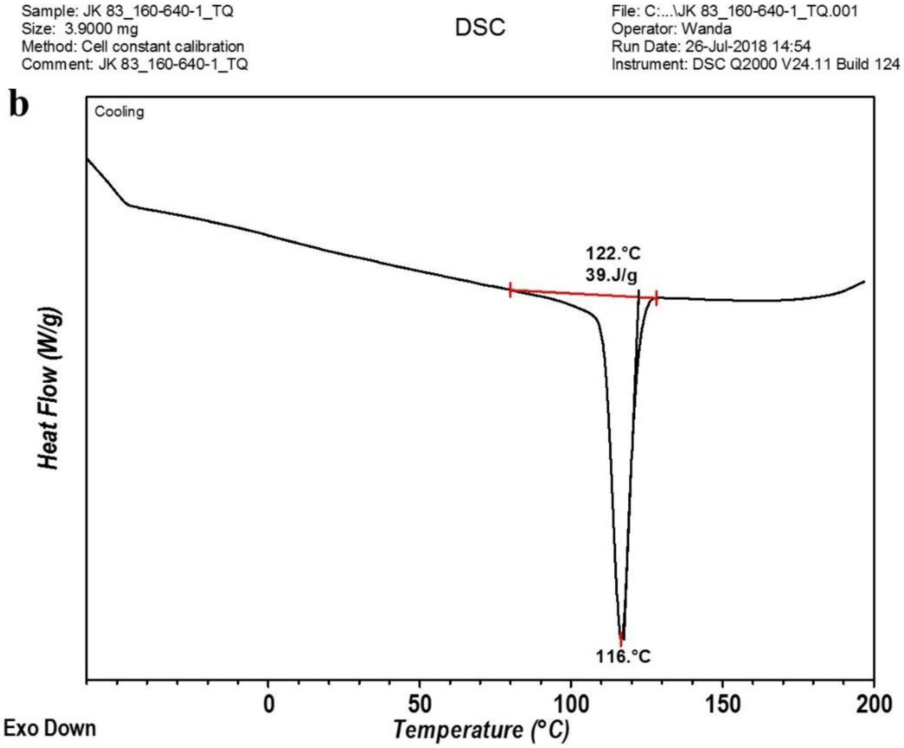
Sample: JK 83_160-640-1_TQ
Size: 3.9000 mg
Method: Cell constant calibration
Comment: JK 83_160-640-1_TQ
DSC
File: C:...\JK 83_160-640-1_TQ.001
Operator: Wanda
Run Date: 26-Jul-2018 14:54
Instrument: DSC Q2000 V24.11 Build 124
Cooling
122.°C
39.J/g
116.°C
Heat Flow (W/g)
0
50
100
150
200
Exo Down
Temperature (°C)

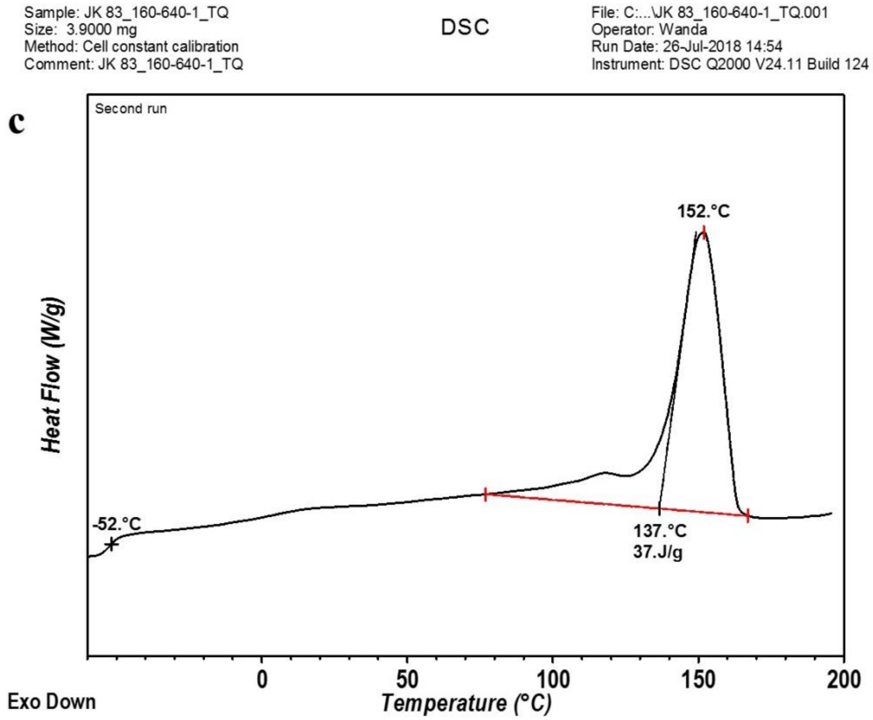

Figure S10. DSC trace a) first run b) cooling c) second run of PDL-*b*-PHB (25 mol% DL) prepared by copolymerization of ε-DL and *rac*-BBL with (salan)Y(III) complexes (Table 3, Entry 5).

a

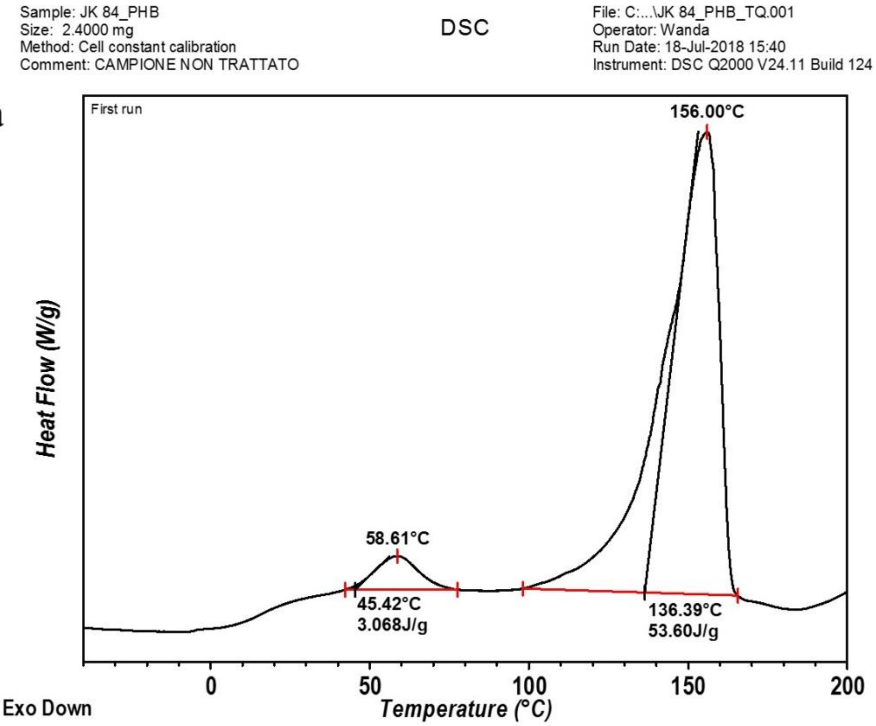
Sample: JK 84_PHB
Size: 2.4000 mg
Method: Cell constant calibration
Comment: CAMPIONE NON TRATTATO
DSC
File: C:...\JK 84_PHB_TQ.001
Operator: Wanda
Run Date: 18-Jul-2018 15:40
Instrument: DSC Q2000 V24.11 Build 124
First run
156.00°C
58.61°C
45.42°C
3.068J/g
136.39°C
53.60J/g
Heat Flow (W/g)
0
50
100
150
200
Exo Down
Temperature (°C)

b

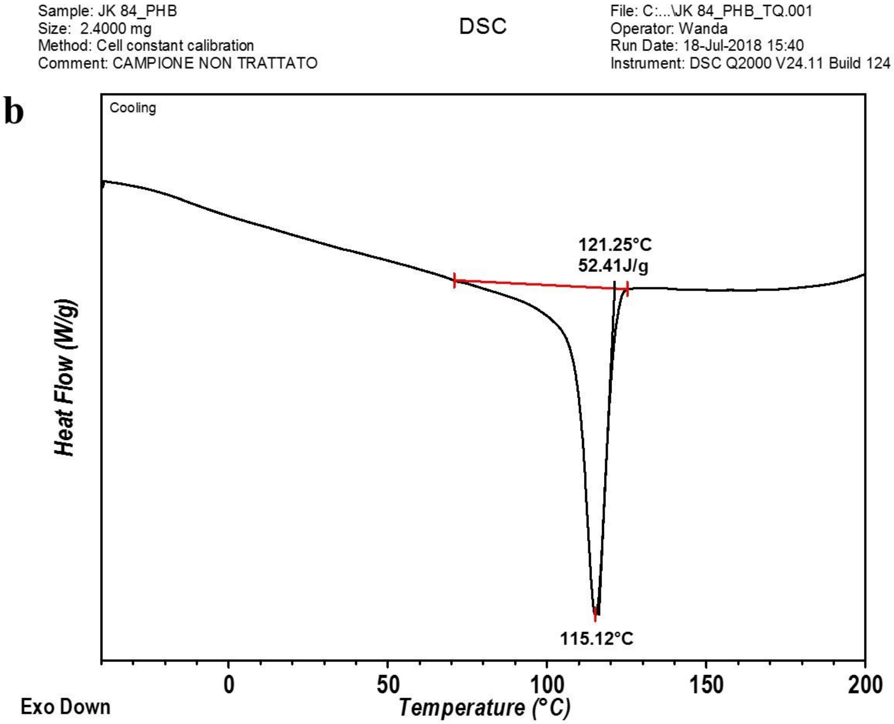
Sample: JK 84_PHB
Size: 2.4000 mg
Method: Cell constant calibration
Comment: CAMPIONE NON TRATTATO
DSC
File: C:...\JK 84_PHB_TQ.001
Operator: Wanda
Run Date: 18-Jul-2018 15:40
Instrument: DSC Q2000 V24.11 Build 124
Cooling
121.25°C
52.41J/g
115.12°C
Heat Flow (W/g)
0
50
100
150
200
Exo Down
Temperature (°C)

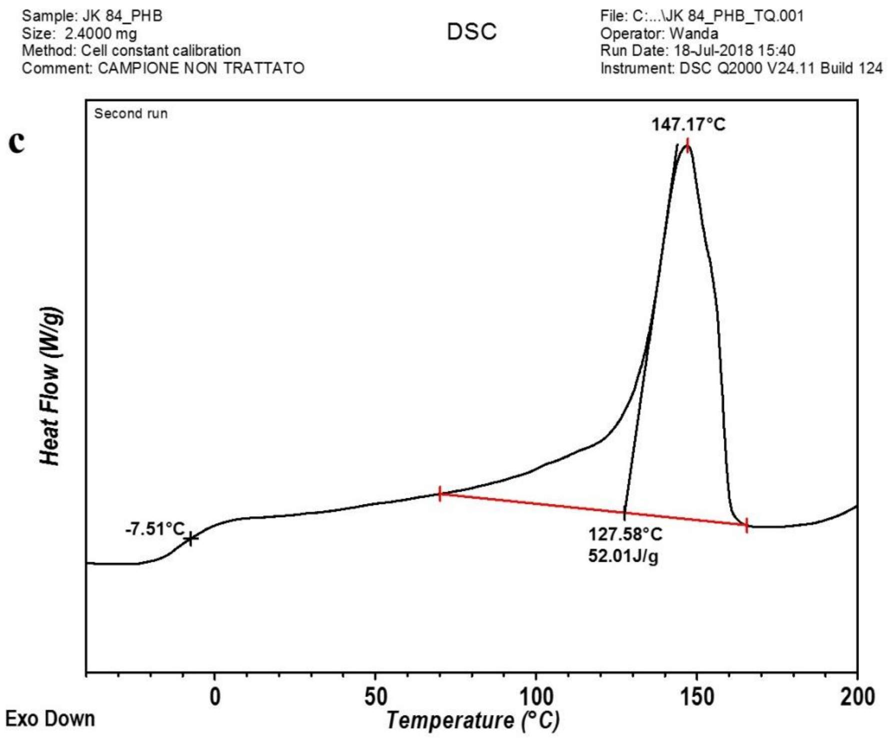

Figure S11. DSC trace a) first run b) cooling c) second run of PHB prepared by ring-opening polymerization of *rac*-BBL with (salan)Y(III) complexes (Table 3, Entry 1).

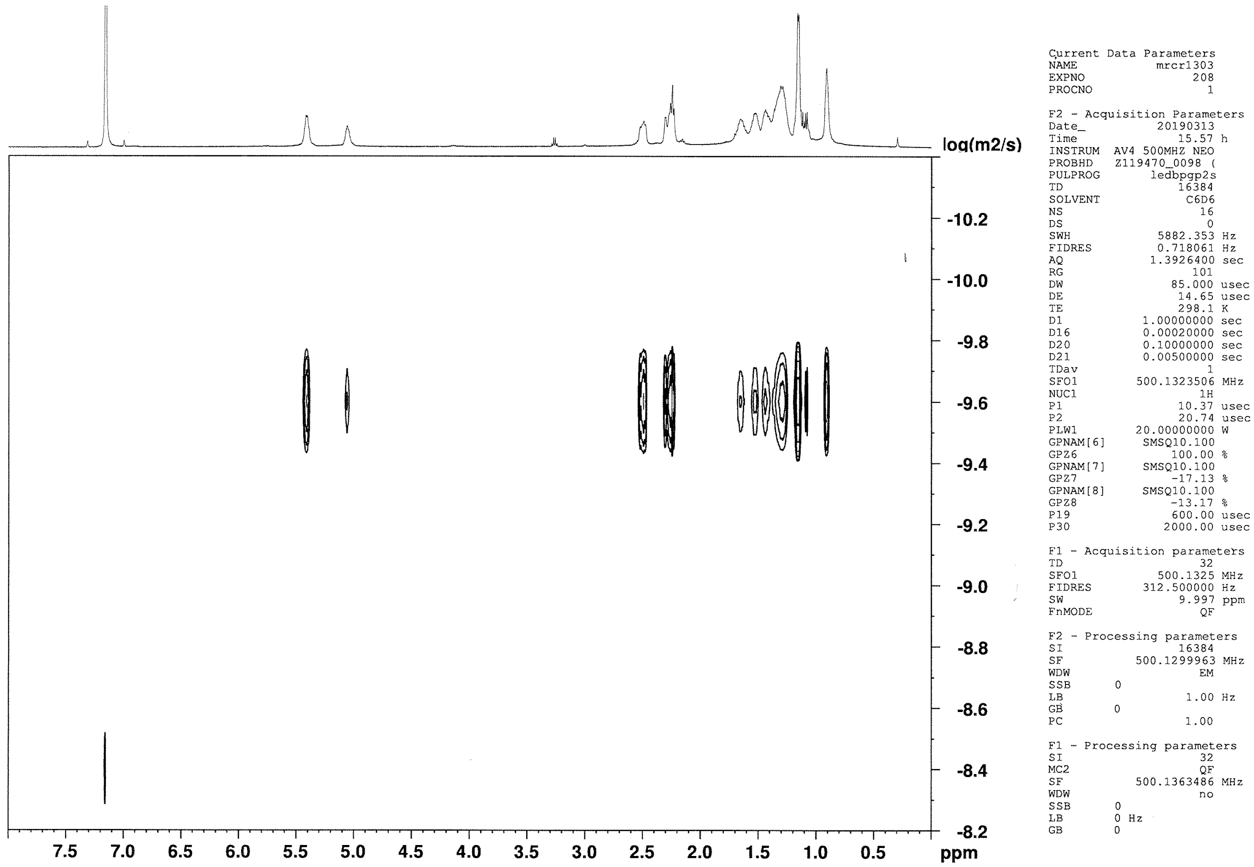

Figure S12. DOSY NMR spectrum of PDL-*b*-PHB in C_6D_6 (Table 2, Entry 1).

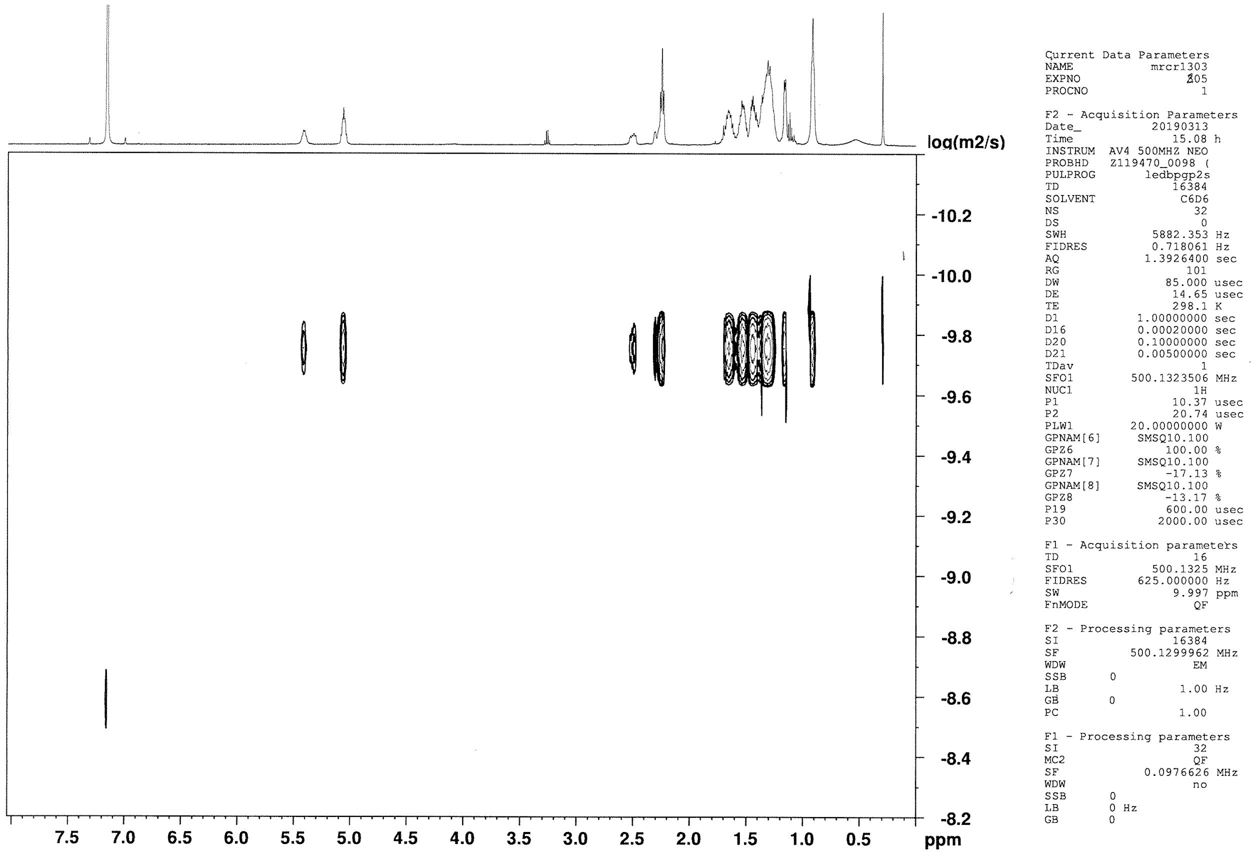

Figure S13. DOSY NMR spectrum of PDL-*b*-PHB in C_6D_6 (Table 2, Entry 7).

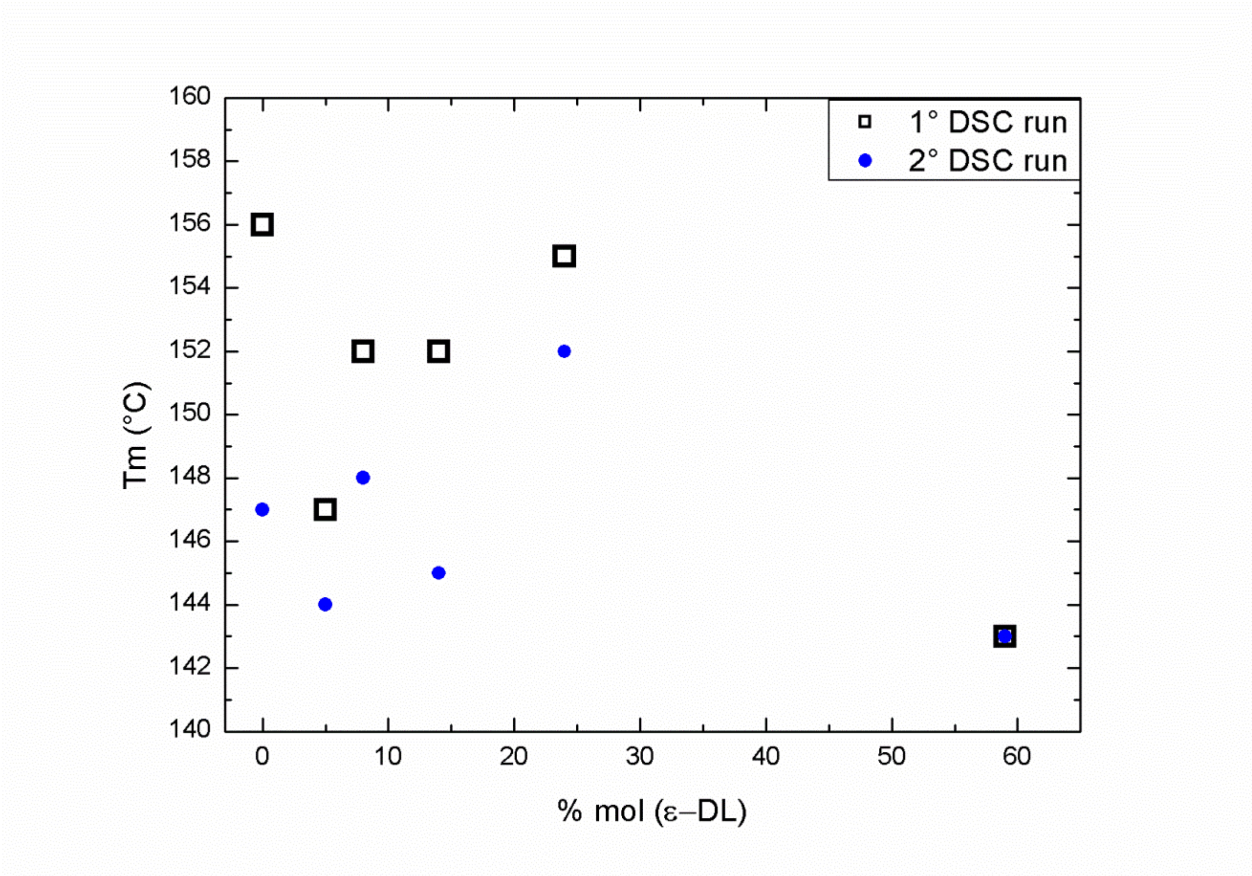

Figure S14. T_m values of both first and second DSC runs, as a function of DL mol%.

REFERENCES

1. Fang, J.; Tschan, M. J.-L.; Roisnel, T.; Trivelli, X.; Gauvin, R. M.; Thomas, C. M.; Maron, L., Yttrium catalysts for syndioselective β-butyrolactone polymerization: on the origin of ligand-induced stereoselectivity. *Polym. Chem.* **2013**, *4*, 360-367.
